# Identifying therapeutic targets for breast cancer: insights from systematic Mendelian randomization analysis

**DOI:** 10.3389/fonc.2024.1407795

**Published:** 2024-06-03

**Authors:** Tao Yao, Yun-Lu Lin, Yu-Qing Wu, Xin-Ge Qian, Zhe-Ning Wang, Sang Qian, Ting Jiang, Jing-Chen Liu, Luo-Xiang Fang, Cheng Zhen, Chun-Hui Wu

**Affiliations:** ^1^ The Second Affiliated Hospital and Yuying Children’s Hospital of Wenzhou Medical University, Wenzhou, Zhejiang, China; ^2^ Children’s Heart Center, The Second Affiliated Hospital and Yuying Children’s Hospital, Institute of Cardiovascular Development and Translational Medicine, Wenzhou Medical University, Wenzhou, Zhejiang, China

**Keywords:** breast cancer, drug targets, Mendelian randomization, single-nucleotide polymorphism, genetic approaches

## Abstract

**Background:**

Breast cancer (BC) exhibits a high incidence rate, imposing a substantial burden on healthcare systems. Novel drug targets are urgently needed for BC. Mendelian randomization (MR) has gained widespread application for identifying fresh therapeutic targets. Our endeavor was to pinpoint circulatory proteins causally linked to BC risk and proffer potential treatment targets for BC.

**Methods:**

Through amalgamating protein quantitative trait loci from 2,004 circulating proteins and comprehensive genome-wide association study data from the Breast Cancer Association Consortium, we conducted MR analyses. Employing Steiger filtering, bidirectional MR, Bayesian colocalization, phenotype scanning, and replication analyses, we further solidified MR study outcomes. Additionally, protein-protein interaction (PPI) network was harnessed to unveil latent associations between proteins and prevailing breast cancer medications. The phenome-wide MR (Phe-MR) was employed to assess potential side effects and indications for the druggable proteins of BC. Finally, we further affirmed the drugability of potential drug targets through mRNA expression analysis and molecular docking.

**Results:**

Through comprehensive analysis, we identified five potential drug targets, comprising four (TLR1, A4GALT, SNUPN, and CTSF) for BC and one (TLR1) for BC_estrogen receptor positive. None of these five potential drug targets displayed reverse causation. Bayesian colocalization suggested that these five latent drug targets shared variability with breast cancer. All drug targets were replicated within the deCODE cohort. TLR1 exhibited PPI with current breast cancer therapeutic targets. Furthermore, Phe-MR unveiled certain adverse effects solely for TLR1 and SNUPN.

**Conclusion:**

Our study uncovers five prospective drug targets for BC and its subtypes, warranting further clinical exploration.

## 1 Introduction

Breast cancer (BC) is the most common malignant tumor of women in clinical practice and seriously endangers women’s physical and mental health. In the year 2020, there were a staggering 2.26 million new cases of BC worldwide, with its incidence having surpassed that of lung cancer to become the global leader (1). With a deepening understanding of BC treatment, a new era of comprehensive treatment has emerged (2). Pharmacological therapy is an indispensable component of the comprehensive treatment approach for BC patients, but it brings about a series of challenges (3). Firstly, the existing medications come with significant side effects (i.e., gastrointestinal reactions, bone marrow suppression, and myocardial structural damage). Secondly, many patients show no response to pharmacological treatment (i.e., treatment-resistant). As such, novel and effective drug therapy targets for BC are still needed.

Proteins play a pivotal role in the pathogenesis and progression of diseases, with circulating proteins often serving as viable targets for pharmacological interventions due to their amenability to direct manipulation. In preceding research, several circulating proteins linked to BC have been documented, including cellular communication network factor 1 (CCN1) (4), serum secreted clusterin (sCLU) (5), and insulin-like growth factor 1 (IGF1) (6). However, most of these studies are observational and yield results that are susceptible to the possibility of confounding or reverse causation bias.

Randomized controlled trials (RCTs) serve as the gold standard for establishing the relationship between drugs (protein targets) and BC. However, the implementation of RCTs is challenged by significant financial and time costs. Mendelian Randomization (MR) is an epidemiological approach that employs genetic variation as a proxy for exposure to predict causal relationships with outcomes (7, 8). Owing to the random allocation of genetic variations during conception, MR is largely impervious to the influence of confounders and reverse causality. The efficacious application of drug-targeted MR analyses has extended to various disorders, such as multiple sclerosis (9), type 1 diabetes (10), and COVID-19 (11). However, there have been limited studies utilizing the drug target MR approach to identify potential targets for BC up to the present.

**Figure 1:**
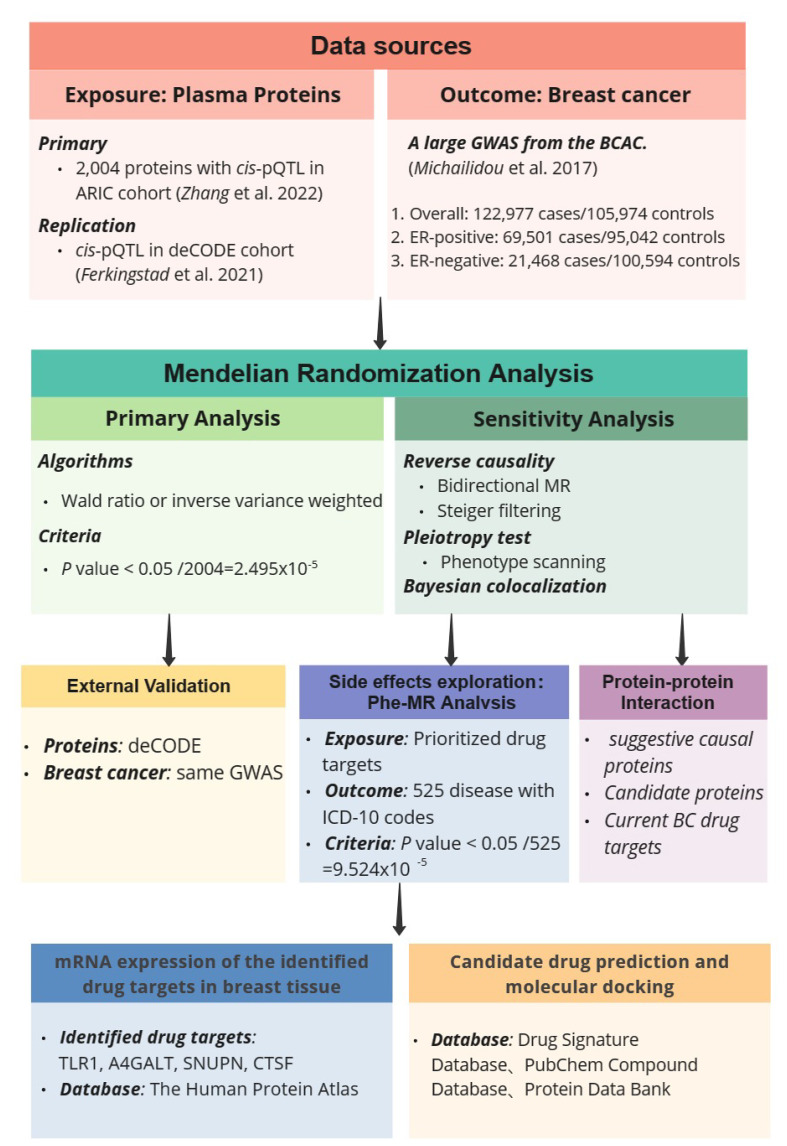
Study design of Mendelian Randomization study to reveal potential drug targets for breast cancer.

In this study, we conducted MR by integrating large-scale BC genome-wide association studies (GWAS) results with cis-protein quantitative trait loci (cis-pQTL) of 2,004 plasma proteins, aiming at identifying plasma proteins that can serve as potential therapeutic targets for BC. To ensure the stability of causal relationships, we conducted a series of sensitivity analyses, including reverse causality detection, pleiotropy test, and Bayesian co-localization analysis. For significant MR results, we utilized cis-pQTL data from the deCODE cohort for replication to validate the preliminary results. In subsequent analyses, we performed protein-protein interaction (PPI) and Gene Ontology (GO) enrichment analyses on suggestive causal proteins (p < 0.05), and visualized a PPI network diagram between prioritized proteins and established BC drug targets. Then, we conducted a phenome-wide MR (Phe-MR) against 525 disease phenotypes to explore side effects. Finally, we further affirmed the drugability of potential drug targets through mRNA expression analysis and molecular docking. The study design is shown in **Figure 1**.

## 2 Materials and methods

All data used in this study were derived from publicly available GWAS statistics and did not require new ethical approval.

### 2.1 Data source

#### 2.1.1 Plasma protein quantitative trait loci

For the primary analysis, the plasma pQTL data were from a recent study by Zhang et al. (12). In summary, Zhang et al. ascertained 2,004 proteins displaying connections with common variants in cis-regions within a populace of 7,213 individuals of European American heritage.

For the replication analysis, we utilized pQTL data in the deCODE cohort by Ferkingstad et al. (13). In this study, Ferkingstad et al. performed GWAS analysis for plasma protein levels (35,559 Icelanders, 4,907 proteins) and found a total of 18,084 associations between sequence variations and plasma protein levels.

#### 2.1.2 Data sources of breast cancer GWAS

We collected genetic association summary statistics for BC risk from the Breast Cancer Association Consortium (BCAC), a meta-analysis of 67 studies including 122,977 cases (thereof 69,501 estrogen receptor (ER)-positive and 21,468 ER-negative), and 105,974 controls for breast cancer (14). Briefly, using the reference panel from the 1000 Genomes Project (phase 3) and adjusting for genetic principal components and country-specific factors, the study evaluated the correlation between breast cancer susceptibility and 11.8 million single-nucleotide polymorphisms (SNPs).

### 2.2 Instrumental variable selection

The SNPs of plasma protein for the primary analysis were filtered according to the following procedures: (i) A genome-wide threshold of significance (p < 5E-08) was adopted. (ii) Based on European ancestry reference data from the 1000 Genomes Project, we employed the PLINK algorithm to clump and discard SNPs (with an r^2^ threshold of 0.001 and a window size of 10000 kB). (iii) Genetic variables with F-statistic <10 were excluded to avoid weak instrumental variable bias (15). (iv) The pQTLs can be divided into cis-acting and trans-acting. Cis-pQTLs exert direct regulation on protein expression at the transcriptional tier, whereas trans-pQTLs govern protein expression via intermediate mechanisms and are susceptible to potential horizontal pleiotropic effects (16). Therefore, only cis-pQTLs were included in our study (**Supplementary Table S1**).

In the replication analysis, we employed both the same-variant strategy and the significant-variant strategy to further validate our findings. The same-variant strategy utilized SNPs identical to those employed in the primary analysis, whereas the significant-variant strategy featured SNPs exhibiting genome-wide significance in the deCODE cohort (**Supplementary Table S2**).

### 2.3 Statistical analysis

#### 2.3.1 Mendelian randomization analysis

To ascertain the causal effects of circulating proteins on BC, two-sample MR analyses were conducted using plasma protein as the exposure and BC as the outcome. In instances of a single available pQTL, we used the Wald ratio method to compute the MR estimates. When two or more instruments were available, the inverse variance-weighted (MR-IVW) approach was employed.

Bonferroni corrections were applied to reduce the false discovery rate. In the primary analysis, given that MR analysis was exclusively conducted for 2004 plasma proteins, the threshold for multiple correction in MR analysis result was set at P < 2.495E-05 (0.05/2004). In the replication analyses, significant thresholds of 0.0045 (0.05/11), 0.025(0.05/2), and 0.0167 (0.05/3) were used for BC_ overall, BC_ER(+), and BC_ER(-).

Causal estimates were presented as odds ratios (ORs) with 95% confidence intervals (CIs) for risk of BC per standard deviation (SD) increase in plasma protein levels. Statistical analyses were performed using the TwoSampleMR package in R 3.4.2.

#### 2.3.2 Sensitivity analyses

Initially, we used Steiger filtering to determine the robustness of the directionality of causality (17). Next, we further performed a bidirectional MR to assess whether there is a reverse causality between plasma protein and BC (18). (**Supplementary Tables S3**-**S5**). Using the same criteria used to screen for pQTL, we extracted instrumental variables from BC GWAS. As Zhang et al.’s study lacked summary statistics for proteins, we obtained the outcome data from the deCODE cohort.

To avoid the impact of pleiotropy, we conducted a search in the phenoscanner database (http://www.phenoscanner.medschl.cam.ac.uk) for all phenotypes associated with the selected instrumental variables. We excluded SNPs (P < 5E -08) linked to BC and any known risk factors of BC.

To further investigate causality of observed MR associations, we performed colocalization analysis between prioritized proteins and BC. This colocalization analysis can determine the probability that the protein level and the risk of BC are affected by the same genetic variants. The analyses were performed using the coloc R package with default parameters, deriving the posterior probabilities of 5 hypotheses (H0-H4) under the Bayesian framework (19). Proteins with high-support evidence of colocalization (PH4> 0.7) were considered as effective drug targets (20).

#### 2.3.3 Protein-protein network

To assess the functional associations and biological processes among MR drug targets, we conducted a PPI analysis and GO enrichment among those proteins with a significance level of p < 0.05. Furthermore, to investigate the relationship between prioritized proteins and known breast cancer drug targets (acquired through literature review (21–24) and DrugBank database (25) search), we performed a PPI analysis between the prioritized proteins and the established breast cancer drug targets. PPI and GO enrichment analyses were constructed by the Search Tool for the Retrieval of Interacting Genes (STRING) database version 12.0 (26) (https://string-db.org/). The visualization of the PPI network was carried out using the Cytoscape platform (https://cytoscape.org/).

#### 2.3.4 Phenome-wide MR analysis

To further explore the potential side effects and more extensive indications of the prioritized proteins screened out in the preliminary analysis, we performed Phe-MR. Specifically, we initially incorporated 525 GWASs from IEU Open GWAS that were conducted within the UK Biobank and defined by ICD-10 diagnostic codes for disease traits (**Supplementary Table S6**). Subsequently, we employed the BC-associated proteins as the exposure and conducted MR analysis with these 525 disease traits as outcomes. If the protein’s effect direction on a particular disease is consistent with its effect direction on BC, it can be inferred that the targeted protein used for treating BC may also confer potential “ benefit “ for that disease. Conversely, if the directions are inconsistent, it indicates the presence of potential adverse effects. The significance level of Phe-MR results was set at P < 9.524E-05 (0.05/525).

### 2.4 mRNA expression analysis of the identified drug targets in different tissues

We performed mRNA expression analysis of the identified drug targets using The Human Protein Atlas database (27). The mRNA expression data is derived from deep sequencing of RNA (RNA-seq) from 40 different normal tissue types, more details about human protein atlas are available in the original publication and the website (proteinatlas.org).

### 2.5 Candidate drug prediction and molecular docking

To assess the drugability of potential drug targets, our study utilized the Drug Signature Database (DSigDB) to predict candidate drugs, and further conducted molecular docking with candidate drugs as ligands and potential drug targets as receptors. DSigDB encompasses 22,527 gene sets and 17,389 distinct compounds, enabling the pairing of clinical drugs with target genes. We uploaded the genes of potential drug targets to DSigDB for candidate drug predictions and obtained the structural data of drugs and proteins from the PubChem Compound Database and the Protein Data Bank, respectively. Subsequently, molecular docking was employed to evaluate the binding affinities and interaction patterns between candidate drugs and targets at the atomic level. Initially, we removed water molecules from ligands and receptors, and introduced polar hydrogen atoms. Afterward, allowing unrestricted molecular movement, appropriately sized pockets were created to envelop all proteins’ structural domains. The process was visualized through AutodockVina 1.2.2.

## 3 Results

### 3.1 Screening the proteome for breast cancer causal proteins

**Figure 2:**
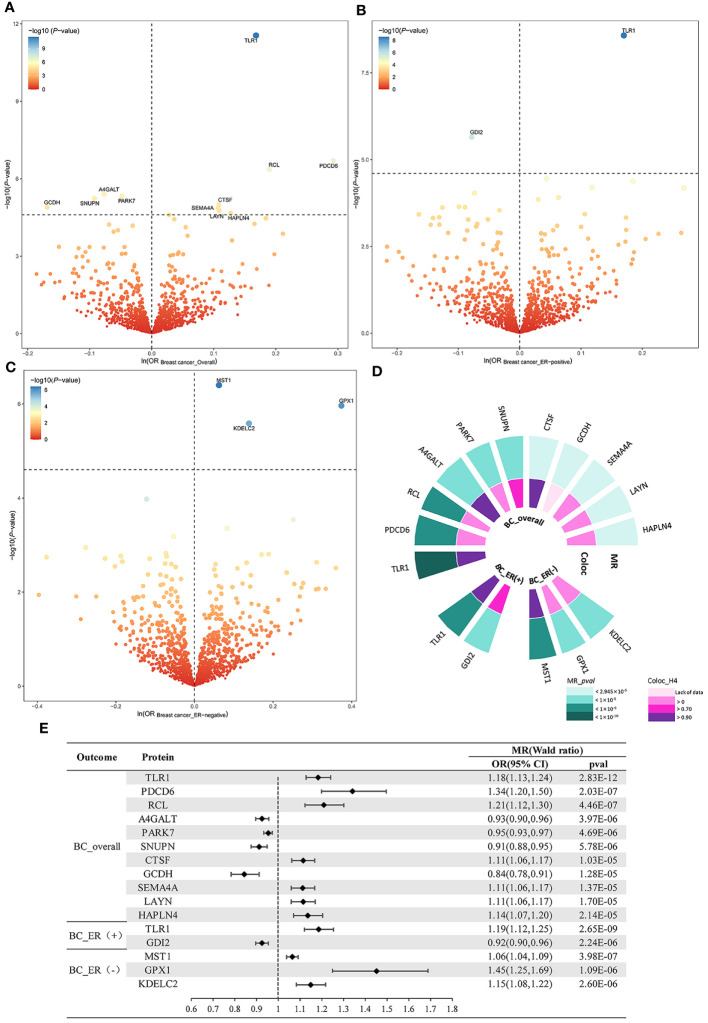
MR and colocalization analysis results in the primary phase. Volcano plots of the MR results for 2004 plasma proteins on the risk of **(A)** BC_overall, **(B)** BC_ER(+), and **(C)** BC_ER(-). **(D)** Pval and value of colocalization analysis for prioritized proteins. **(E)** Forest plot for the MR result between pQTL and BC. Dashed horizontal black line corresponded to P = 2.495E-05 (0.05/2004). ln = natural logarithm.

Genetic instruments of plasma proteins for MR discovery analysis are shown in **Supplementary Table S1**. The MR analysis yielded 11 BC_overall-related proteins, 2 BC_ER(+)-related proteins, and 3 BC_ER(-)-related proteins respectively at a Bonferroni corrected threshold (P < 2.495E-05)(**Table 1**, **Figures 2A–C**). To be specific, increased Lactosylceramide 4-alpha-galactosyltransferase (A4GALT) (OR = 0.93; 95% CI, 0.90–0.96; P = 3.97E-06), Protein DJ-1 (PARK7) (OR = 0.95; 95% CI, 0.93–0.97; P = 4.69E-06), Snurportin-1 (SNUPN) (OR = 0.91; 95% CI, 0.88–0.95; P = 5.78E-06), and Glutaryl-CoA dehydrogenase, mitochondrial (GCDH) (OR = 0.84; 95% CI, 0.78–0.91; P = 1.28E-05) decreased the risk of BC_overall, whereas elevated Toll-like receptor 1 (TLR1) (OR = 1.18; 95% CI, 1.13–1.24; P = 2.83E-12), Programmed cell death protein 6 (PDCD6) (OR = 1.34; 95% CI, 1.20–1.50; P = 2.03E-07), 2’-deoxynucleoside 5’-phosphate N-hydrolase 1 (RCL) (OR = 1.21; 95% CI, 1.12–1.30; P = 4.46E-07), Cathepsin F (CTSF) (OR = 1.11; 95% CI, 1.06–1.17; P = 1.03E-05), Semaphorin-4A (SEMA4A) (OR = 1.11; 95% CI, 1.06–1.17; P = 1.37E-05), Layilin (LAYN) (OR = 1.11; 95% CI, 1.06–1.17; P = 1.70E-05), and Hyaluronan and proteoglycan link protein 4 (HAPLN4) (OR = 1.14; 95% CI, 1.07–1.20; P = 2.14E-05) increased the risk of BC_overall. Rab GDP dissociation inhibitor beta (GDI2) (OR = 0.92; 95% CI, 0.90–0.96; P = 2.24E-06) was associated with a lower risk of BC_ER(+), while TLR1 (OR = 1.19; 95% CI, 1.12–1.25; P = 2.65E-09) was associated with a higher risk of BC_ER(+). Higher genetically predicted levels of Hepatocyte growth factor-like protein (MST1) (OR = 1.06; 95% CI, 1.04–1.09; P = 3.98E-07), Glutathione peroxidase 1 (GPX1) (OR = 1.45; 95% CI, 1.25–1.69; P = 1.09E-06), and KDEL motif-containing protein 2 (KDELC2) (OR = 1.15; 95% CI, 1.08–1.22; P = 2.60E-06) were all associated with higher risk of BC_ER(-) (**Figures 2D, E**).

**Table 1:** Summary of primary analysis, reverse causality detection, phenotype scanning, Bayesian colocalization analysis, and replication analysis on the prioritized proteins.

Outcome	Protein	UniProt ID	SNP	MR(Wald ratio)		Steiger filtering (pval)	Bidirectional MR		Phenotype scanning	Bayesian colocalization PPH4	Replication analysis(significant)	Replication analysis(same)	Evidence of potential drug target
				OR(95% CI)	pval		OR(95% CI)	pval					
BC_overall	TLR1	Q15399	rs5743618	1.18(1.13,1.24)	2.83E-12	1.07E-46 Passed	1.025(0.986,1.066)	0.216 Passed	Passed	0.977(YES)	2.83E-12 Passed	2.83E-12 Passed	**YES**
	PDCD6	O75340	rs56075848	1.34(1.20,1.50)	2.03E-07	2.94E-09 Passed	1.005(0.981,1.030)	0.673 Passed	Passed	3.283E-04(NO)	1.39E-05 Passed	2.03E-07 Passed	NO
	RCL	O43598	rs114371775	1.21(1.12,1.30)	4.46E-07	9.10E-21 Passed	1.011(0.987,1.035)	0.383 Passed	Passed	0.562(NO)	3.47E-05 Passed	*	NO
	A4GALT	Q9NPC4	rs8138197	0.93(0.90,0.96)	3.97E-06	7.83E-104 Passed	0.992(0.969,1.015)	0.482 Passed	Passed	0.960(YES)	3.40E-06 Passed	3.97E-06 Passed	**YES**
	PARK7	Q99497	rs17523802	0.95(0.93,0.97)	4.69E-06	2.18E-188 Passed	1.011(0.988,1.034)	0.353 Passed	Passed	0.698(NO)	0.0168 NO	4.69E-06 Passed	NO
	SNUPN	O95149	rs7170787	0.91(0.88,0.95)	5.78E-06	1.01E-71 Passed	0.994(0.972,1.017)	0.606 Passed	Passed	0.729(YES)	1.23E-05 Passed	5.78E-06 Passed	**YES**
	CTSF	Q9UBX1	rs1044522	1.11(1.06,1.17)	1.03E-05	4.81E-47 Passed	1.008(0.982,1.035)	0.548 Passed	Passed	0.916(YES)	7.53E-06 Passed	1.03E-05 Passed	**YES**
	GCDH	Q92947	rs2238641	0.84(0.78,0.91)	1.28E-05	6.56E-20 Passed	Lack of data		Passed	Lack of data			NO
	SEMA4A	Q9H3S1	rs12401997	1.11(1.06,1.17)	1.37E-05	1.54E-50 Passed	1.007(0.982,1.034)	0.585 Passed	Passed	0.116(NO)	2.21E-05 Passed	1.37E-05 Passed	NO
	LAYN	Q6UX15	rs4938792	1.11(1.06,1.17)	1.70E-05	3.26E-44 Passed	1.014(0.989,1.039)	0.268 Passed	Passed	0.512(NO)	1.70E-05 Passed	1.70E-05 Passed	NO
	HAPLN4	Q86UW8	rs55762233	1.14(1.07,1.20)	2.14E-05	9.37E-30 Passed	1.011(0.984,1.038)	0.438 Passed	Passed	2.120E-05(NO)	1.00E-05 Passed	2.14E-05 Passed	NO
BC_ER(+)	TLR1	Q15399	rs5743618	1.19(1.12,1.25)	2.65E-09	3.74E-46 Passed	1.028(0.983,1.075)	0.232 Passed	Passed	0.948(YES)	2.65E-09 Passed	2.65E-09 Passed	**YES**
	GDI2	P50395	rs55913768	0.92(0.90,0.96)	2.24E-06	2.89E-150 Passed	1.006(0.983,1.030)	0.583 Passed	Passed	0.789(NO)	Lack of data	2.24E-06 Passed	NO
BC_ER (-)	MST1	P26927	rs3197999	1.06(1.04,1.09)	3.98E-07	1.89E-178 Passed	0.995(0.970,1.021)	0.688 Passed	NO	0.958(YES)	1.23E-06 Passed	3.98E-07 Passed	NO
	GPX1	P07203	rs9823546	1.45(1.25,1.69)	1.09E-06	1.87E-14 Passed	0.996(0.964,1.029)	0.801 Passed	NO	0.282(NO)	Lack of data	1.09E-06 Passed	NO
	KDELC2	Q7Z4H8	rs141379009	1.15(1.08,1.22)	2.60E-06	7.52E-168 Passed	0.999(0.927,1.075)	0.971 Passed	Passed	0.652(NO)	2.29E-06 Passed	2.60E-06 Passed	NO

Odds ratios per SD increase in plasma protein levels as BC and its subtypes risk increased. CI, confidence level; MR, Mendelian randomization; PPH4, posterior probability of hypothesis 4; SNP, single-nucleotide polymorphism. In Steiger filtering, a P <0.05 suggests no reverse causality, while in bidirectional MR, a P >0.05 also suggests no reverse causality. *indicates the exclusion of SNPs with incompatible alleles during replication analysis, rendering the analysis unfeasible.

### 3.2 Sensitivity analysis for breast cancer causal proteins

#### 3.2.1 Reverse causality detection

Steiger filtering analysis substantiated the accurate causal direction from protein levels to the development of BC for the 15 proteins prioritized in this study. Meanwhile, Bidirectional MR analysis did not reveal any causal effect of BC on the 15 prioritized proteins (**Table 1**, **Supplementary Figure S1**).Phenome-wide MR analysis.

**Figure 3:**
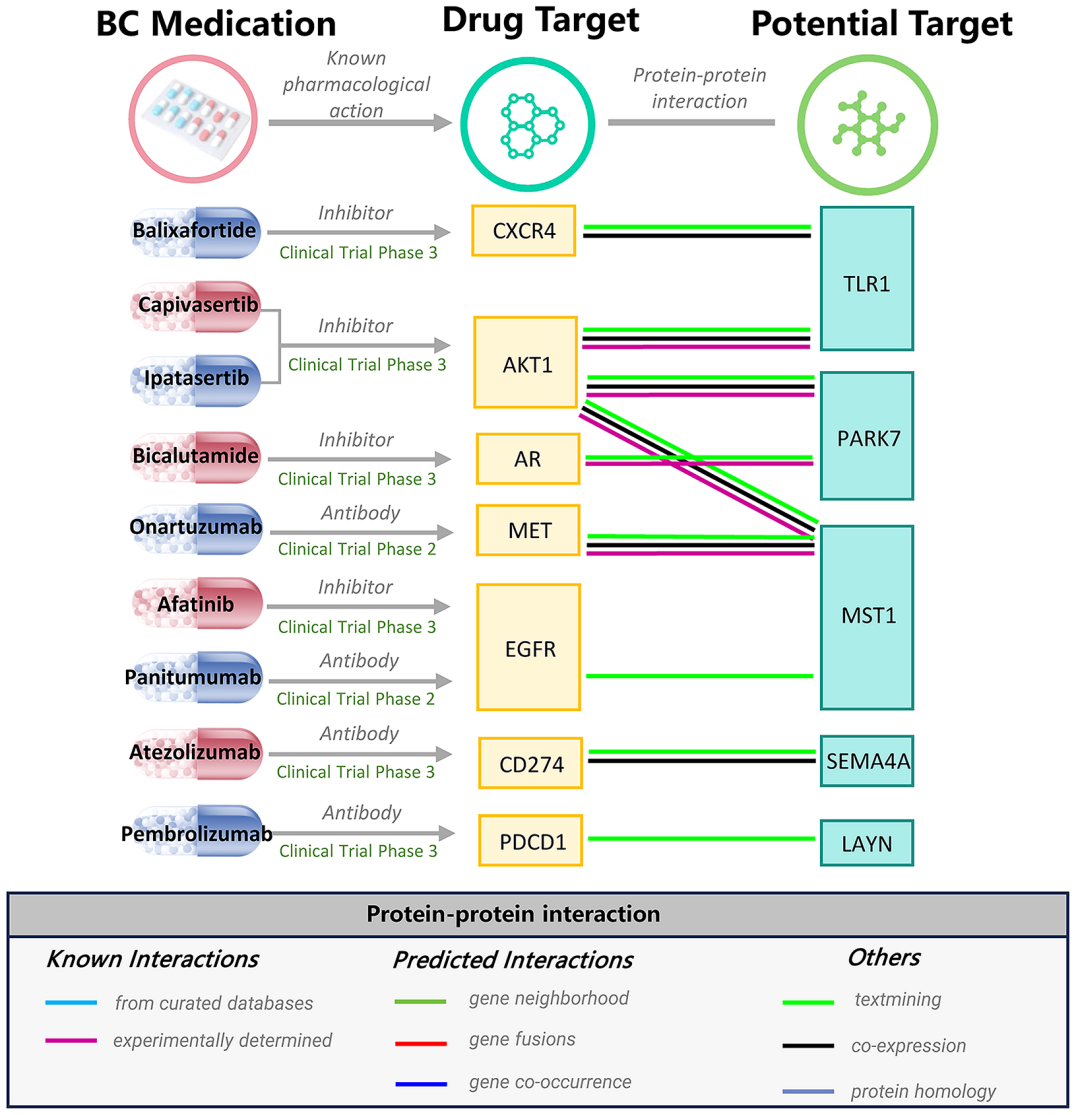
Interaction between current BC medications targets and prioritized proteins.

A total of 525 diseases were included in the Phenome-wide MR analysis (**Supplementary Table S10** and **Figure 3**). Under Bonferroni correction (P < 0.05/525 = 9.5 × 10^-5^), an increase in CCM levels was associated with a reduced risk of colorectal malignancy, while BTN3A3 exhibited associations with multiple diseases, including hyperplasia of prostate, unspecified hematuria, inguinal hernia, and hypothyroidism(**Supplementary Tables S11**-**21**). Analysis of the other nine plasma proteins did not reveal significant side effects.

#### 3.2.2 Phenotype scanning

Phenoscanner revealed some associations between SNP and other diseases or proteins. Specifically, we observed associations of genetic instruments for TLR1 and PARK7 with certain allergic diseases such as allergic asthma, allergic rhinitis, and eczema. Genetic instruments for PARK7, GPX1, and MST1 were linked to various digestive system disorders including sclerosing cholangitis and inflammatory bowel disease. This might suggest shared etiology between BC and the mentioned diseases. Importantly, we found MST1 and GPX1 to be associated with multiple proteins, hence excluding them from potential drug targets (**Table 1**, **Supplementary Table S7**).

#### 3.2.3 Bayesian co-localization analysis

To delve deeper into the causality of the detected MR associations, we performed colocalization analyses of prioritized protein with BC and its subtype outcomes. To be specific, BC_overall had high support for colocalization with 4 proteins including TLR1 (PPH4 = 0.977), A4GALT (PPH4 = 0.960), SNUPN (PPH4 = 0.729), and CTSF (PPH4 = 0.916). For BC_ER(+), we found both TLR1 (PPH4 = 0.977) and GDI2 (PPH4 = 0.789) with strong supporting colocalization evidence. For BC_ER(-), only MST1 (PPH4 = 0.958) passed the test. Summarily, six potentially druggable proteins with evidence of a causative genetic variant between the pQTL and BC risk were identified from colocalization analyses (**Table 1**, **Figure 2D**, **Supplementary Figures S2**-**S16**).

### 3.3 PPI network and GO enrichment analysis

We observed the complicated PPI networks of the suggestive significant MR proteins for BC and its subtypes, all of which were significantly enriched (p < 1.0E-16, p < 1.0E-16, and p = 1.76E-11) (**Supplementary Figures S17**-**S19**). Meanwhile, PPI analysis showed that APOE and STAT3 seem to play a pivotal role in all proteins and are strongly associated with the development of breast cancer. GO enrichment analysis revealed that the suggestive significant MR proteins for BC_overall, BC_ER (+), and BC_ER (-) were significantly enriched in “sulfur compound metabolic process”, “ small molecule metabolic process” and “ response to chemical”, respectively (**Supplementary Tables S8**-**S10**).

The PPI network unveiled connections involving five prioritized proteins with the targets of seven existing medications for BC, including 5 robust interactions (AKT1-TLR1, AKT1-PARK7, AKT1-MST1, AR-PARK7, and MET-MST1) (**Figure 3**). Specifically, TLR, PARK7, and MST1 are associated with serine/threonine kinase 1 (AKT1), which serves as the target for ipatasertib and capivasertib. Bicalutamide is an androgen receptor (AR) inhibitor, and AR is related to PARK7. MST1 is linked to Hepatocyte growth factor receptor (MET), which is the target of Onartuzumab. The above correlation further strengthens the possibility of our priority protein as a drug target.

### 3.4 Phe-MR analysis of the side effects of breast cancer causal proteins

**Figure 4:**
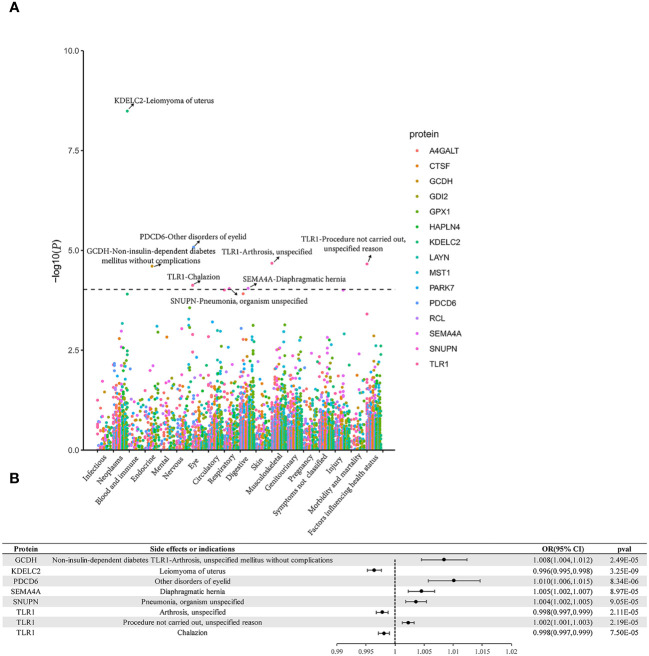
Phe-MR analysis results. **(A)** Manhattan plot. **(B)** Forest plot. Dashed horizontal black line corresponded to P = 9.524E-05 (0.05/525). A dot represents a disease trait, and different colors represent the MR result of different proteins.

Phe-MR findings indicated that some proteins held potential as favorable drug targets for alternate indications. For instance, while genetically- proxied TLR1 elevation raises the risk of BC_overall and BC_ER(+), it concurrently heightens the likelihood of non-performance of surgery or procedure (negative treatment outcome). Genetically determined SEMA4A elevation escalated the risk of both BC_overall and diaphragmatic hernia. Elevated genetic levels of plasma PDCD6 were linked to an elevated risk of BC_overall, as well as some disorders of eyelid. When considering the mentioned proteins as potential drug targets for breast cancer, they may also exert beneficial effects on the corresponding diseases or traits mentioned above. In contrast, gene-predicted TLR1 was linked to arthrosis and chalazion, gene-predicted SNUPN was linked to pneumonia, gene-predicted KDELC2 was linked to uterine leiomyoma, and gene-predicted GCDH was linked to non-insulin-dependent diabetes mellitus without complications, all of which were considered detrimental. These unfavorable effects need to be taken into consideration when evaluating their potential as preventive utility for BC (**Figure 4**, **Supplementary Tables S11**-**S25**).

### 3.5 External replication of breast cancer causal proteins

We attempted to replicate the effect estimates for the prioritized proteins using data from the deCODE cohort. In the significant-variant strategy, PARK7 (P=0.017) failed to be successfully replicated, and due to a lack of data, GCDH, GDI2, and GPX1 were also not replicated. In the same-variant strategy, RCL and GCDH were not successfully replicated due to a lack of data and the presence of incompatible alleles (**Supplementary Figure S20**).

### 3.6 mRNA expression of the identified drug targets in breast tissue

We searched for identified drug targets in The Human Protein Atlas database and observed that the mRNA expression levels of these identified drug targets rank among the top tissues in breast tissue compared to other tissues throughout the body. This further corroborates the pharmacological potential of the identified targets (**Supplementary Figures S21**-**S24**).

### 3.7 Candidate drug prediction and Molecular docking

**Figure 5:**
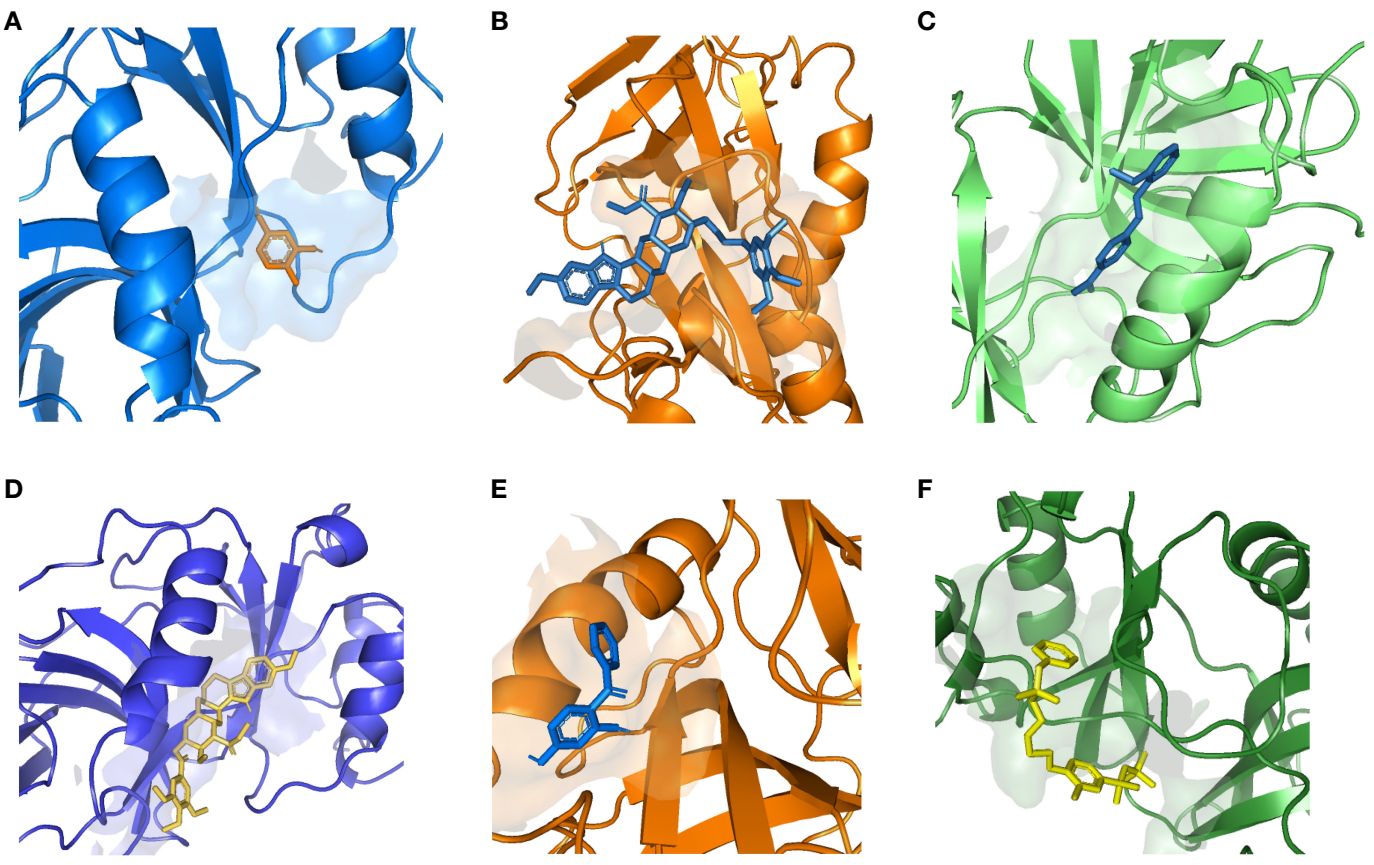
Docking results of available proteins with small molecules. **(A)** SNUPN docking 5-Amino-2-methylphenol, **(B)** SNUPN docking Recinnamine, **(C)** SNUPN docking Pinaflavol, **(D)** SNUPN docking reserpine, **(E)** SNUPN docked to 2,4-DIHYDROXYBENZOPHENONE, **(F)** SNUPN docked to Methylbenzethonium chloride.

Based on adjusted p-values and searches in DSigDB, we identified a total of 10 available compounds (**Supplementary Table S26**). Due to the lack of protein structure data, we were unable to complete the molecular docking for A4GALT with chlorophyllin. However, it is noteworthy that SNUPN exhibited remarkably stable binding with candidate drugs, suggesting robust affinity and providing compelling evidence for its druggability (**Figure 5**, **Supplementary Table S27**).

## 4 Discussion

Despite the steady progress in breast cancer treatment approaches, breast cancer therapy still faces numerous challenges. Therefore, the development of new drugs for breast cancer is urgently needed. In this study, based on a large-scale pQTL dataset, we identified 15 prioritized proteins that may influence BC outcomes [11 for BC_overall, 3 for BC_ER(+), and 2 for BC_ER(-)], 12 of which were also found in a repeated study. To mitigate reverse causality, horizontal pleiotropy, or genetic confounding due to linkage disequilibrium (LD), we conducted a series of sensitivity analyses to strengthen our conclusions. Reverse causality detection was performed using Steiger filtering and Bidirectional MR analysis. Encouragingly, we did not identify any proteins with reverse causal effects on BC. Next, phenoscanner was used to detect horizontal pleiotropy. The genetic instruments of MST1 and GPX1 appear to be associated with a variety of proteins, suggesting that MST1 and GPX1 might function as hub proteins regulating multiple pathways. Therefore, we temporarily excluded MST1 and GPX1 from the list of candidate drug targets due to their complex biological functions. Phenoscanner also revealed associations between SNP and several allergic diseases and digestive system disorders. Finally, Bayesian colocalization was employed to assess whether the MR findings were impacted by linkage disequilibrium. Out of the 16 potential targets, 7 (43.75%) potential targets passed the colocalization test with 0.7 as the critical threshold for posterior probability. All in all, through pleiotropy scanning, reverse causality detection, colocalization analysis, and replication analysis, we further identified five potential drug targets: TLR1, A4GALT, SNUPN, and CTSF for BC_overall and TLR1 for BC_ER(+). Utilizing a multifaceted approach, including GO enrichment analysis, PPI analysis, molecular docking simulations, and mRNA expression analysis, we have extended the confirmation of the pharmacological viability of the predicted target proteins. Last but not least, the Phe-MR indicated a few potential safety concerns.

TLR1 is a member of the Toll-like receptors (TLRs) family, expressed on the surface of immune cells. TLRs are integral components of the innate immune system, crucial for protecting the host against bacterial and viral infections. Emerging evidence indicates that the TLRs/NF-κB signaling cascade assumes a substantial role in the etiology and recidivism of BC (28). Upon engagement with either endogenous or exogenous ligands, TLRs stimulate intracellular signaling pathways, culminating in the release of diverse cytokines, including tumor necrosis factor-alpha and interleukin-1, which orchestrate chronic inflammatory responses. This activation cascade in turn triggers NF-κB signaling, potentiating tumor cell proliferation. Moreover, González-Reyes et al., through immunohistochemistry, protein blotting, and real-time PCR analysis, identified a significant increase in the mRNA levels of TLR3, TLR4, and TLR9 in recurrent breast cancer samples (29). These findings align with our discoveries, underscoring the potential promise of TLR1 antagonists in BC treatment. However, the potential latent off-target effects, such as inadvertent immunosuppression, cast uncertainty on the feasibility of small molecule TLR1 antagonists. As evident in our phe-MR study, adverse effects like arthrosis and chalazion could be mediated by immune suppression. Furthermore, our PPI analysis has unveiled an intricate interplay between TLR1 and CXCR4, as well as AKT1. Notably, CXCR4 has been identified as the therapeutic target of Balixafortide, whereas AKT1 as the therapeutic target of Capivasertib and Ipatasertib, all of which have been subjected to rigorous scrutiny in phase III clinical trials for breast cancer. This compelling convergence of evidence amplifies the hypothesis that TLR1 stands as a promising novel therapeutic avenue for BC intervention.

Snurportin 1, also known as SNUPN, is a protein involved in the transport of small nuclear ribonucleoproteins between the cytoplasm and the nucleus of a cell. Historically, research on SNUPN has been limited, although there is a more extensive study of a protein that collaborates with SNUPN, known as exportin 1 (XPO1). SNUPN and XPO1 mutually recognize each other, forming a nuclear pore complex or a cargo together, leading to abnormal cellular localization of oncogenes, tumor suppressor genes, and signaling pathway mediators. This disruption of cellular homeostasis contributes to the initiation and progression of tumorigenesis, particularly in the context of leukemia (30). A recent proteome association study has further confirmed the association between SNUPN and breast cancer, which was in consistent with our finding (31).

Cathepsin F is a member of the Cathepsin family, playing a pivotal role in protein degradation and metabolic processes within cells. Research indicates that Cathepsin F may be associated with the development and progression of certain tumors, such as lung cancer (32), thyroid cancer (33), and cervical cancer (34), although there is a lack of studies investigating its relationship with breast cancer. Other members of the cathepsin protease family have been reported to have connections with breast cancer. For example, Vashum Y et al. indicated that CTSK is specifically associated with breast cancer bone metastasis by promoting adipocyte differentiation (35). Additionally, Cathepsin D has been found to be upregulated and secreted by breast cancer cells, promoting tumor invasion and metastasis by degrading the extracellular matrix and basement membrane in an acidic environment (36). Based on the aforementioned studies, there is reason to believe that the Cathepsin family plays a significant role in the occurrence and progression of breast cancer. Therefore, CTSF as a potential therapeutic target for breast cancer is credible.

Lactosylceramide 4-alpha-galactosyltransferase, encoded by the A4GALT gene in humans, participates in the pathway of sphingolipid metabolism, specifically responsible for attaching a galactose moiety to lactosylceramide molecules, thereby forming an α-1,4-galactose linkage (37). Despite the limited extent of previous investigation into 4AGALT, we postulate that the sphingolipid metabolism mediated by 4AGALT could intricately modulate the composition and structure of cell membranes, consequently exerting influence over the tumor microenvironment. Both our MR and co-localization analyses robustly affirm the association between A4GALT and breast cancer. However, further research is requisite to elucidate the underlying mechanistic basis of this relationship.

This study possesses numerous merits. Primarily, it is widely recognized that the drug development process inherently demands substantial time, exorbitant costs, and encounters a notable risk of failure. Employing drug targeting MR, through an analysis of vast-scale biological data encompassing the genome and proteome, pertinent plasma proteins associated with BC were identified, thereby pinpointing potential drug targets and amplifying the efficiency of drug development. Additionally, the MR method obviates confounding and reverse causality inherent in observational studies. Thirdly, complementary analyses such as co-localization analysis, phe-MR, drug targets PPI analysis, and replication analysis contribute to the comprehensiveness and reliability of our research findings.

Still, this study is not without certain limitations. Firstly, our analysis was exclusively focused on individuals of European descent, posing challenges in extrapolating the findings to other ancestral groups. Furthermore, while interventions targeting circulating plasma proteins may exert systemic impacts, the attainment of precise modulation within specific tissues remains uncertain. Of paramount importance, it is noteworthy that MR analysis does not entirely recapitulate clinical trials, as patient responses to pharmaceutical interventions inherently manifest diversity within clinical practice. Consequently, clinical trials are warranted to meticulously evaluate the preliminary efficacy and safety profile of these latent drug targets for BC intervention.

In conclusion, this study embraced a thorough genetic methodology to evaluate the intricate interrelationship of plasma proteins with BC and its subcategories. It is noteworthy to emphasize that our study findings underscore the feasibility of TLR1, A4GALT, SNUPN, and CTSF as viable therapeutic targets for BC_overall or BC_ER(+) subtypes. Subsequent investigations hold the promise of corroborating our observations and delving into the underlying mechanisms that warrant exploration.

## Data availability statement

The original contributions presented in the study are included in the article/**Supplementary Material**. Further inquiries can be directed to the corresponding author.

## Author contributions

TY: Writing – original draft, Conceptualization. YL: Writing – original draft, Conceptualization. YW: Writing – original draft, Conceptualization. XQ: Writing – review & editing, Data curation. ZW: Writing – review & editing, Data curation. SQ: Writing – review & editing, Data curation. TJ: Software, Writing – review & editing. JL: Writing – review & editing, Software. LF: Writing – review & editing, Software. CZ: Writing – review & editing, Software. CW: Writing – original draft, Conceptualization.

## References

[1] Sung H Ferlay J Siegel RL Laversanne M Soerjomataram I Jemal A. Global cancer statistics 2020: globocan estimates of incidence and mortality worldwide for 36 cancers in 185 countries. CA Cancer J Clin. (2021) 71:209–49. doi: 10.3322/caac.21660 33538338

[2] Tong CWS Wu M Cho WCS To KKW. Recent advances in the treatment of breast cancer. Front Oncol. (2018) 8:227. doi: 10.3389/fonc.2018.00227 29963498 PMC6010518

[3] Alvarez RH. Present and future evolution of advanced breast cancer therapy. Breast Cancer Res. (2010) 12 Suppl 2:S1. doi: 10.1186/bcr2572 PMC2972555 21050422

[4] Bartkowiak K Heidrich I Kwiatkowski M Banys-Paluchowski M Andreas A Wurlitzer M. Circulating cellular communication network factor 1 protein as a sensitive liquid biopsy marker for early detection of breast cancer. Clin Chem. (2022) 68:344–53. doi: 10.1093/clinchem/hvab153 34458901

[5] Chen QF Chang L Su Q Zhao Y Kong B. Clinical importance of serum secreted clusterin in predicting invasive breast cancer and treatment responses. Bioengineered. (2021) 12:278–85. doi: 10.1080/21655979.2020.1868732 PMC8806267 33356806

[6] Schernhammer ES Holly JM Pollak MN Hankinson SE. Circulating levels of insulin-like growth factors, their binding proteins, and breast cancer risk. Cancer Epidemiol Biomarkers Prev. (2005) 14:699–704. doi: 10.1158/1055-9965.EPI-04-0561 15767352

[7] Hingorani A Humphries S. Nature's randomised trials. Lancet. (2005) 366:1906–8. doi: 10.1016/S0140-6736(05)67767-7 16325682

[8] Skrivankova VW Richmond RC Woolf BAR Davies NM Swanson SA VanderWeele TJ. Strengthening the reporting of observational studies in epidemiology using mendelian randomisation (Strobe-mr): explanation and elaboration. BMJ. (2021) 375:n2233. doi: 10.1136/bmj.n2233 34702754 PMC8546498

[9] Lin J Zhou J Xu Y. Potential drug targets for multiple sclerosis identified through mendelian randomization analysis. Brain. (2023) 146:3364–72. doi: 10.1093/brain/awad070 PMC10393411 36864689

[10] Yazdanpanah N Yazdanpanah M Wang Y Forgetta V Pollak M Polychronakos C. Clinically relevant circulating protein biomarkers for type 1 diabetes: evidence from a two-sample mendelian randomization study. Diabetes Care. (2022) 45:169–77. doi: 10.2337/dc21-1049 34758976

[11] Gaziano L Giambartolomei C Pereira AC Gaulton A Posner DC Swanson SA. Actionable druggable genome-wide mendelian randomization identifies repurposing opportunities for covid-19. Nat Med. (2021) 27:668–76. doi: 10.1038/s41591-021-01310-z PMC7612986 33837377

[12] Zhang J Dutta D Kottgen A Tin A Schlosser P Grams ME. Plasma proteome analyses in individuals of european and african ancestry identify cis-pqtls and models for proteome-wide association studies. Nat Genet. (2022) 54:593–602. doi: 10.1038/s41588-022-01051-w 35501419 PMC9236177

[13] Ferkingstad E Sulem P Atlason BA Sveinbjornsson G Magnusson MI Styrmisdottir EL. Large-scale integration of the plasma proteome with genetics and disease. Nat Genet. (2021) 53:1712–21. doi: 10.1038/s41588-021-00978-w 34857953

[14] Michailidou K Lindstrom S Dennis J Beesley J Hui S Kar S. Association analysis identifies 65 new breast cancer risk loci. Nature. (2017) 551:92–4. doi: 10.1038/nature24284 PMC5798588 29059683

[15] Shim H Chasman DI Smith JD Mora S Ridker PM Nickerson DA. A multivariate genome-wide association analysis of 10 ldl subfractions, and their response to statin treatment, in 1868 caucasians. PloS One. (2015) 10:e0120758. doi: 10.1371/journal.pone.0120758 25898129 PMC4405269

[16] Swerdlow DI Kuchenbaecker KB Shah S Sofat R Holmes MV White J. Selecting instruments for mendelian randomization in the wake of genome-wide association studies. Int J Epidemiol. (2016) 45:1600–16. doi: 10.1093/ije/dyw088 PMC5100611 27342221

[17] Hemani G Tilling K Davey Smith G. Orienting the causal relationship between imprecisely measured traits using gwas summary data. PloS Genet. (2017) 13:e1007081. doi: 10.1371/journal.pgen.1007081 29149188 PMC5711033

[18] Davey Smith G Hemani G. Mendelian randomization: genetic anchors for causal inference in epidemiological studies. Hum Mol Genet. (2014) 23:R89–98. doi: 10.1093/hmg/ddu328 PMC4170722 25064373

[19] Giambartolomei C Vukcevic D SChadt EE Franke L Hingorani AD Wallace C. Bayesian test for colocalisation between pairs of genetic association studies using summary statistics. PloS Genet. (2014) 10:e1004383. doi: 10.1371/journal.pgen.1004383 24830394 PMC4022491

[20] Chen L Peters JE Prins B Persyn E Traylor M Surendran P. Systematic mendelian randomization using the human plasma proteome to discover potential therapeutic targets for stroke. Nat Commun. (2022) 13:6143. doi: 10.1038/s41467-022-33675-1 36253349 PMC9576777

[21] Liang Y Zhang H Song X Yang Q. Metastatic heterogeneity of breast cancer: molecular mechanism and potential therapeutic targets. Semin Cancer Biol. (2020) 60:14–27. doi: 10.1016/j.semcancer.2019.08.012 31421262

[22] Li Y Zhang H Merkher Y Chen L Liu N Leonov S. Recent advances in therapeutic strategies for triple-negative breast cancer. J Hematol Oncol. (2022) 15:121. doi: 10.1186/s13045-022-01341-0 36038913 PMC9422136

[23] Dieras V Campone M Yardley DA Romieu G Valero V Isakoff SJ. Randomized, phase ii, placebo-controlled trial of onartuzumab and/or bevacizumab in combination with weekly paclitaxel in patients with metastatic triple-negative breast cancer. Ann Oncol. (2015) 26:1904–10. doi: 10.1093/annonc/mdv263 26202594

[24] Emens LA. Breast cancer immunotherapy: facts and hopes. Clin Cancer Res. (2018) 24:511–20. doi: 10.1158/1078-0432.CCR-16-3001 PMC5796849 28801472

[25] Wishart DS Feunang YD Guo AC Lo EJ Marcu A Grant JR. Drugbank 5.0: a major update to the drugbank database for 2018. Nucleic Acids Res. (2018) 46:D1074–82. doi: 10.1093/nar/gkx1037 PMC5753335 29126136

[26] Szklarczyk D Gable AL Lyon D Junge A Wyder S Huerta-Cepas J. String V11: protein-protein association networks with increased coverage, supporting functional discovery in genome-wide experimental datasets. Nucleic Acids Res. (2019) 47:D607–D13. doi: 10.1093/nar/gky1131 PMC6323986 30476243

[27] Uhlen M Fagerberg L Hallstrom BM Lindskog C Oksvold P Mardinoglu A. Proteomics. Tissue-based map of the human proteome. Science. (2015) 347:1260419. doi: 10.1126/science.1260419 25613900

[28] Bhatelia K Singh K Singh R. Tlrs: linking inflammation and breast cancer. Cell Signal. (2014) 26:2350–7. doi: 10.1016/j.cellsig.2014.07.035 25093807

[29] Gonzalez-Reyes S Marin L Gonzalez L Gonzalez LO del Casar JM Lamelas ML. Study of tlr3, tlr4 and tlr9 in breast carcinomas and their association with metastasis. BMC Cancer. (2010) 10:665. doi: 10.1186/1471-2407-10-665 21129170 PMC3009680

[30] Jain P Kanagal-Shamanna R Wierda W Keating M Sarwari N Rozovski U. Clinical and molecular characteristics of xpo1 mutations in patients with chronic lymphocytic leukemia. Am J Hematol. (2016) 91:E478–E9. doi: 10.1002/ajh.24496 PMC5073031 27468087

[31] Gregga I Pharoah PDP Gayther SA Manichaikul A Im HK Kar SP. Predicted proteome association studies of breast, prostate, ovarian, and endometrial cancers implicate plasma protein regulation in cancer susceptibility. Cancer Epidemiol Biomarkers Prev. (2023) 32:1198–207. doi: 10.1158/1055-9965.EPI-23-0309 PMC10528410 37409955

[32] Wei S Liu W Xu M Qin H Liu C Zhang R. Cathepsin F and fibulin-1 as novel diagnostic biomarkers for brain metastasis of non-small cell lung cancer. Br J Cancer. (2022) 126:1795–805. doi: 10.1038/s41416-022-01744-3 PMC9174239 35217799

[33] Wang Y Mei J Zhang Y He X Zheng X Tan J. Cathepsin F genetic mutation is associated with familial papillary thyroid cancer. Am J Med Sci. (2022) 364:414–24. doi: 10.1016/j.amjms.2022.03.017 35447134

[34] Vazquez-Ortiz G Pina-Sanchez P Vazquez K Duenas A Taja L Mendoza P. Overexpression of Cathepsin F, Matrix metalloproteinases 11 and 12 in cervical cancer. BMC Cancer. (2005) 5:68. doi: 10.1186/1471-2407-5-68 15989693 PMC1175083

[35] Vashum Y Khashim Z. Obesity and cathepsin K: A complex pathophysiological relationship in breast cancer metastases. Endocr Metab Immune Disord Drug Targets. (2020) 20:1227–31. doi: 10.2174/1871530320666200505115132 32368981

[36] Ashraf Y Mansouri H Laurent-Matha V Alcaraz LB Roger P Guiu S. Immunotherapy of triple-negative breast cancer with cathepsin D-targeting antibodies. J Immunother Cancer. (2019) 7:29. doi: 10.1186/s40425-019-0498-z 30717773 PMC6360707

[37] Stenfelt L Westman JS Hellberg A Olsson ML. The P1 histo-blood group antigen is present on human red blood cell glycoproteins. Transfusion. (2019) 59:1108–17. doi: 10.1111/trf.15115 30597575

