# Supplementary material for: Identifying therapeutic targets for breast cancer: insights from systematic Mendelian randomization analysis
Source: Front Oncol. 2024 Jun 3;14:1407795. doi: 10.3389/fonc.2024.1407795 (PMC11180768; doi:10.3389/fonc.2024.1407795)
Supplement: Supplementary file 1 [file Image_1.pdf]

Supplementary Material

Identifying Therapeutic Targets for Breast Cancer: Insights from Systematic Mendelian Randomization Analysis

Tao Yao [1†], Yun-Lu Lin [1†], Yu-Qing Wu [1], Xin-Ge Qian [1], Zhe-Ning Wang [1], Sang Qian [1], Ting Jiang [1], Jing-Chen Liu [1], Luo-Xiang Fang [1], Cheng Zhen [1], and Chun-Hui Wu [2*]

[1]The Second Affiliated Hospital and Yuying Children's Hospital of Wenzhou Medical University, Wenzhou 325000, Zhejiang, China

[2]Children's Heart Center, The Second Affiliated Hospital and Yuying Children's Hospital, Institute of Cardiovascular Development and Translational Medicine, Wenzhou Medical University, Wenzhou, 325027, Zhejiang, China

†These authors contributed equally to this work and share first authorship

*** Correspondence:**
Chun-Hui Wu
19817585212@163.com

Supplementary Figures

Figure S1 Bidirectional MR analysis for breast cancer on levels of prioritized proteins

Figure S2 Bayesian colocalization analysis for TLR1 and BC_overall

Figure S3 Bayesian colocalization analysis for PDCD6 and BC_overall

Figure S4 Bayesian colocalization analysis for RCL and BC_overall

Figure S5 Bayesian colocalization analysis for A4GALT and BC_overall

Figure S6 Bayesian colocalization analysis for PARK7 and BC_overall

Figure S7 Bayesian colocalization analysis for SNUPN and BC_overall

Figure S8 Bayesian colocalization analysis for CTSF and BC_overall

Figure S9 Bayesian colocalization analysis for SEMA4A and BC_overall

Figure S10 Bayesian colocalization analysis for LAYN and BC_overall

Figure S11 Bayesian colocalization analysis for HAPLN4 and BC_overall

Figure S12 Bayesian colocalization analysis for TLR1 and BC_ER(+)

Figure S13 Bayesian colocalization analysis for GDI2 and BC_ ER(+)

Figure S14 Bayesian colocalization analysis for MST1 and BC_ ER(-)

Figure S15 Bayesian colocalization analysis for GPX1 and BC_ ER(-)

Figure S16 Bayesian colocalization analysis for KDELC2 and BC_ ER(-)

Figure S17 Protein-protein interaction network between suggestive BC_overall-related proteins ($P < 0.05$) from plasma

Figure S18 Protein-protein interaction network between suggestive BC_ER(+)-related proteins ($P < 0.05$) from plasma

Figure S19 Protein-protein interaction network between suggestive BC_ER(-)-related proteins ($P < 0.05$) from plasma

Figure S20 Replication of causal relationship between prioritized proteins and breast cancer

Figure S21 The mRNA expression profile of TLR1 across human tissues (The human protein atlas, https://www.proteinatlas.org/).

Figure S22 The mRNA expression profile of A4GALT across human tissues (The human protein atlas, https://www.proteinatlas.org/).

Figure S23 The mRNA expression profile of SNUPN across human tissues (The human protein atlas, https://www.proteinatlas.org/).

Figure S24 The mRNA expression profile of CTSF across human tissues (The human protein atlas, https://www.proteinatlas.org/).

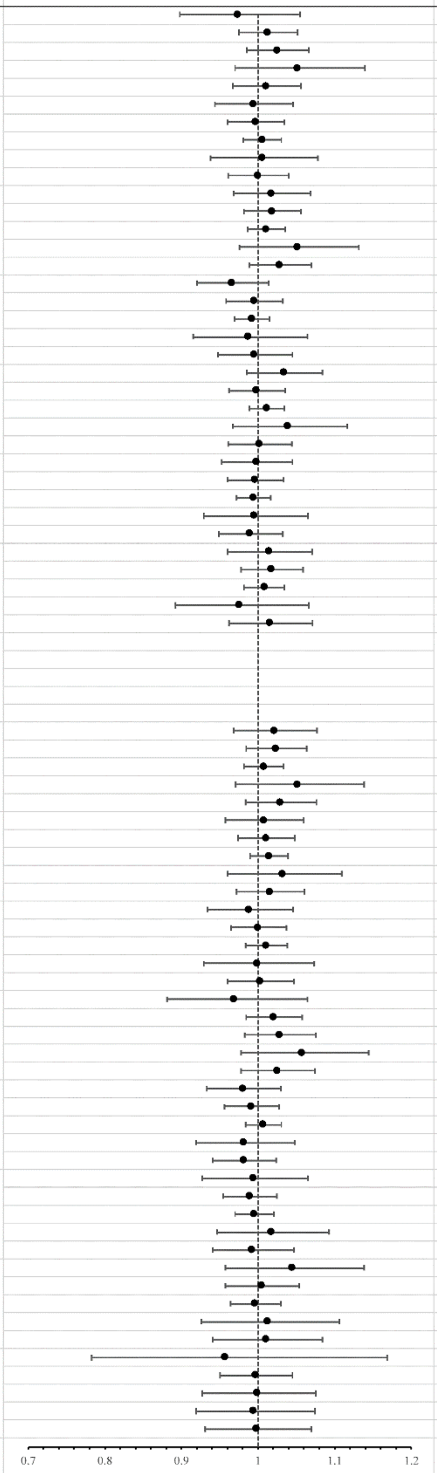

Exposure	Protein	method	nsnp	OR(95% CI)	pval
BC_overall	TLR1	MR Egger	129	0.973(0.898,1.055)	0.510
		Weighted median	129	1.012(0.975,1.051)	0.532
		Inverse variance weighted	129	1.025(0.986,1.066)	0.216
		Simple mode	129	1.051(0.97,1.139)	0.225
		Weighted mode	129	1.01(0.967,1.056)	0.643
	PDCD6	MR Egger	129	0.993(0.944,1.046)	0.800
		Weighted median	129	0.997(0.96,1.034)	0.859
		Inverse variance weighted	129	1.005(0.981,1.03)	0.673
		Simple mode	129	1.005(0.938,1.078)	0.879
		Weighted mode	129	1(0.961,1.04)	0.982
	RCL	MR Egger	129	1.017(0.968,1.068)	0.504
		Weighted median	129	1.018(0.982,1.056)	0.336
		Inverse variance weighted	129	1.011(0.987,1.035)	0.383
		Simple mode	129	1.051(0.976,1.132)	0.192
		Weighted mode	129	1.028(0.989,1.069)	0.166
	A4GALT	MR Egger	129	0.966(0.92,1.014)	0.166
		Weighted median	129	0.995(0.958,1.032)	0.778
		Inverse variance weighted	129	0.992(0.969,1.015)	0.482
		Simple mode	129	0.987(0.916,1.065)	0.741
		Weighted mode	129	0.995(0.948,1.045)	0.849
	PARK7	MR Egger	129	1.034(0.986,1.084)	0.173
		Weighted median	129	0.998(0.962,1.036)	0.916
		Inverse variance weighted	129	1.011(0.988,1.034)	0.353
		Simple mode	129	1.039(0.967,1.116)	0.300
		Weighted mode	129	1.001(0.961,1.044)	0.944
	SNUPN	MR Egger	129	0.998(0.953,1.045)	0.928
		Weighted median	129	0.996(0.96,1.034)	0.834
		Inverse variance weighted	129	0.994(0.972,1.017)	0.606
		Simple mode	129	0.994(0.929,1.065)	0.874
		Weighted mode	129	0.989(0.948,1.032)	0.616
	CTSF	MR Egger	129	1.014(0.96,1.071)	0.618
		Weighted median	129	1.017(0.978,1.058)	0.400
		Inverse variance weighted	129	1.008(0.982,1.035)	0.548
		Simple mode	129	0.975(0.892,1.066)	0.582
		Weighted mode	129	1.015(0.962,1.071)	0.579
	GCDH	Lack of data			
	SEMA4A	MR Egger	129	1.021(0.968,1.077)	0.449
		Weighted median	129	1.023(0.984,1.063)	0.251
		Inverse variance weighted	129	1.007(0.982,1.034)	0.585
		Simple mode	129	1.052(0.971,1.139)	0.217
		Weighted mode	129	1.029(0.984,1.077)	0.213
	LAYN	MR Egger	129	1.007(0.957,1.06)	0.777
		Weighted median	129	1.01(0.974,1.047)	0.592
		Inverse variance weighted	129	1.014(0.989,1.039)	0.268
		Simple mode	129	1.032(0.96,1.109)	0.393
		Weighted mode	129	1.015(0.972,1.061)	0.490
	HAPLN4	MR Egger	129	0.988(0.934,1.046)	0.684
		Weighted median	129	1(0.965,1.037)	1.000
		Inverse variance weighted	129	1.011(0.984,1.038)	0.438
		Simple mode	129	0.999(0.929,1.073)	0.969
		Weighted mode	129	1.003(0.96,1.047)	0.898
BC_ER（+）	TLR1	MR Egger	93	0.968(0.881,1.064)	0.507
		Weighted median	93	1.02(0.984,1.058)	0.272
		Inverse variance weighted	93	1.028(0.983,1.075)	0.232
		Simple mode	93	1.057(0.977,1.144)	0.168
		Weighted mode	93	1.025(0.977,1.075)	0.314
	GDI2	MR Egger	93	0.98(0.933,1.029)	0.419
		Weighted median	93	0.991(0.956,1.027)	0.626
		Inverse variance weighted	93	1.006(0.983,1.03)	0.583
		Simple mode	93	0.981(0.919,1.048)	0.575
		Weighted mode	93	0.981(0.941,1.024)	0.384
BC_ER（-）	MST1	MR Egger	31	0.994(0.927,1.065)	0.864
		Weighted median	31	0.989(0.955,1.025)	0.547
		Inverse variance weighted	31	0.995(0.97,1.021)	0.688
		Simple mode	31	1.017(0.947,1.093)	0.647
		Weighted mode	31	0.992(0.941,1.046)	0.776
	GPX1	MR Egger	31	1.044(0.957,1.139)	0.338
		Weighted median	31	1.004(0.957,1.054)	0.861
		Inverse variance weighted	31	0.996(0.964,1.029)	0.801
		Simple mode	31	1.012(0.926,1.106)	0.791
		Weighted mode	31	1.01(0.941,1.084)	0.785
	KDELC2	MR Egger	31	0.957(0.783,1.169)	0.667
		Weighted median	31	0.997(0.95,1.045)	0.889
		Inverse variance weighted	31	0.999(0.927,1.075)	0.971
		Simple mode	31	0.994(0.919,1.074)	0.875
		Weighted mode	31	0.998(0.931,1.069)	0.946

Figure S1 Bidirectional MR analysis for breast cancer on levels of prioritized proteins

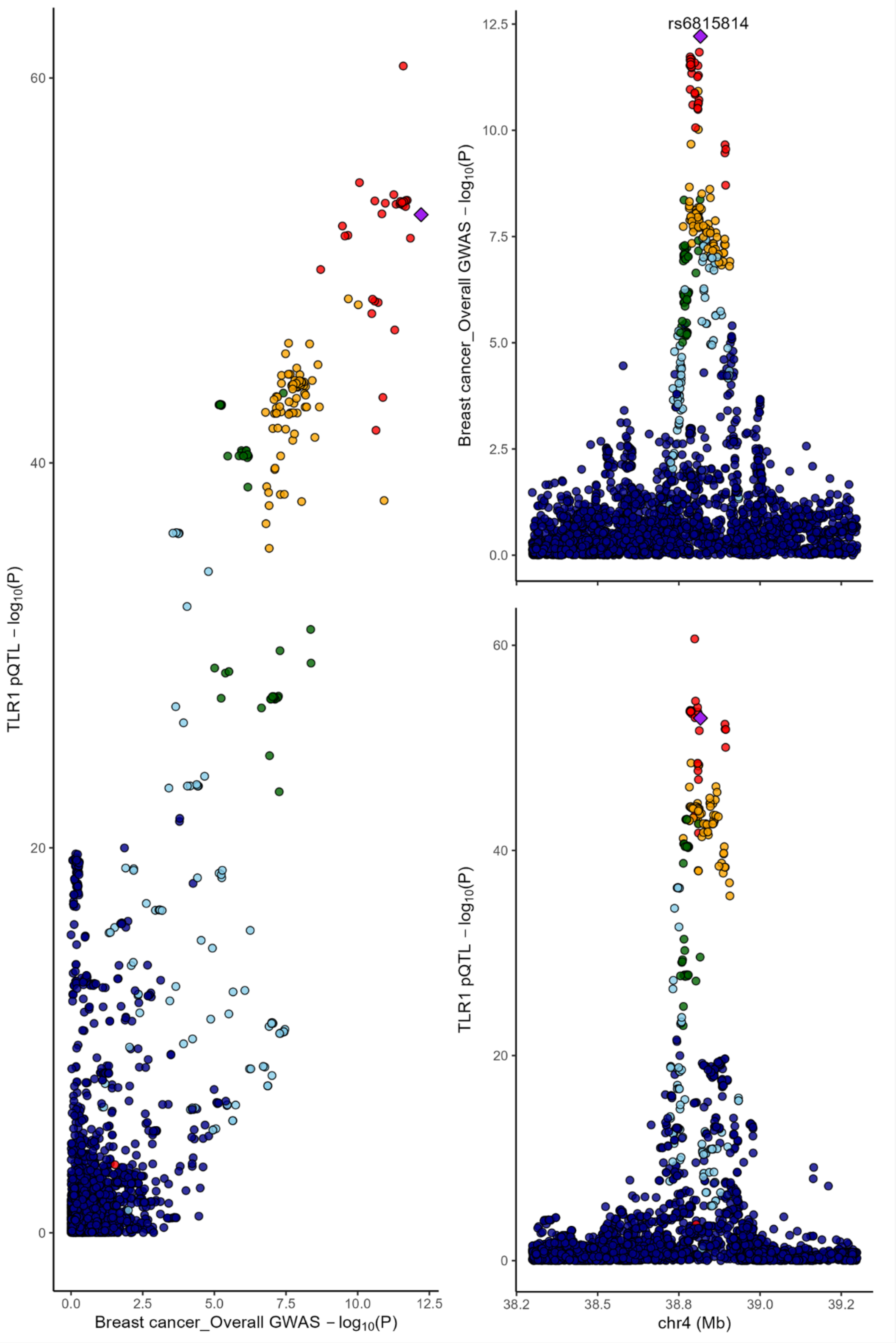

Figure S2 Bayesian colocalization analysis for TLR1 and BC_overall

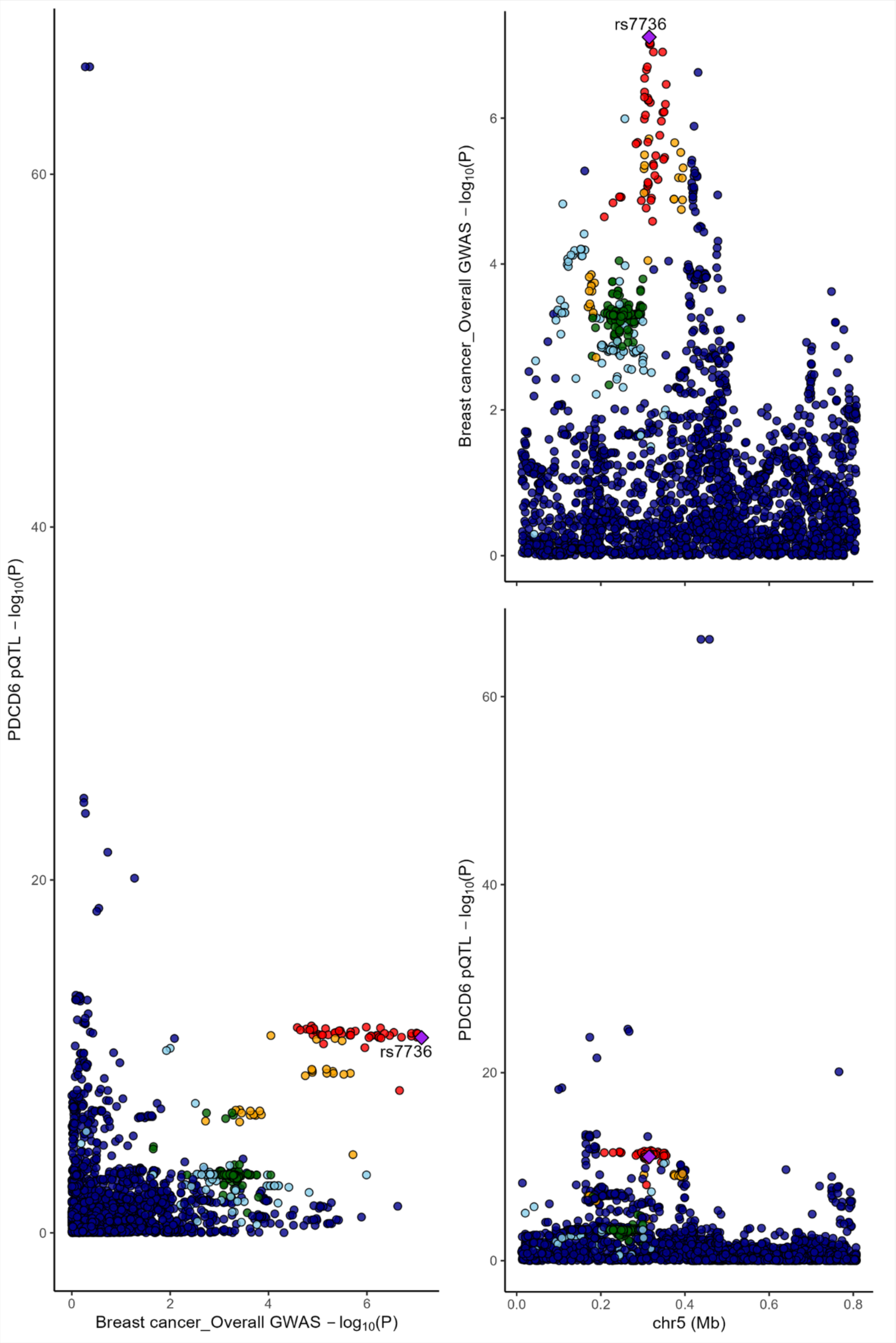

Figure S3 Bayesian colocalization analysis for PDCD6 and BC_overall

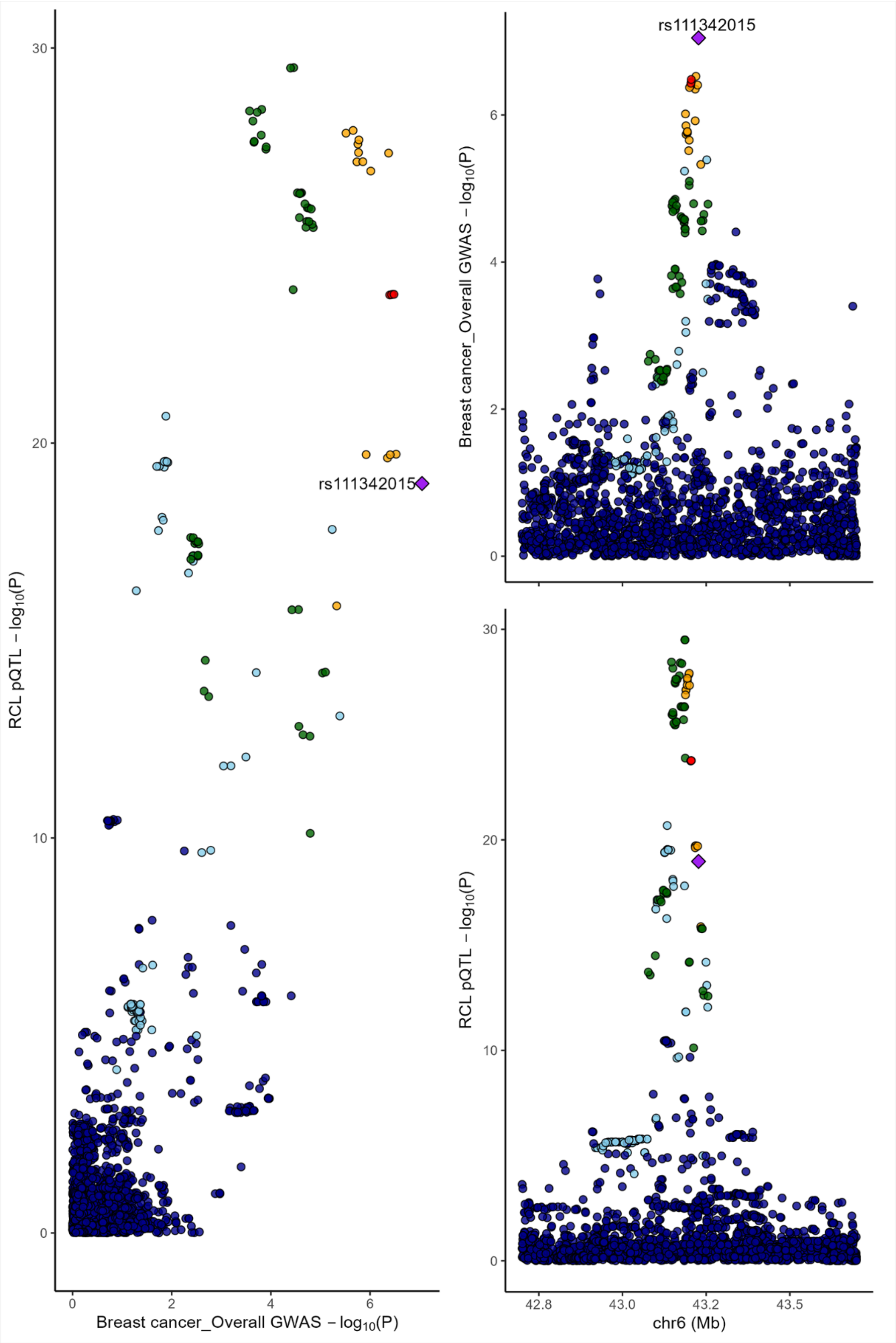

Figure S4 Bayesian colocalization analysis for RCL and BC_overall

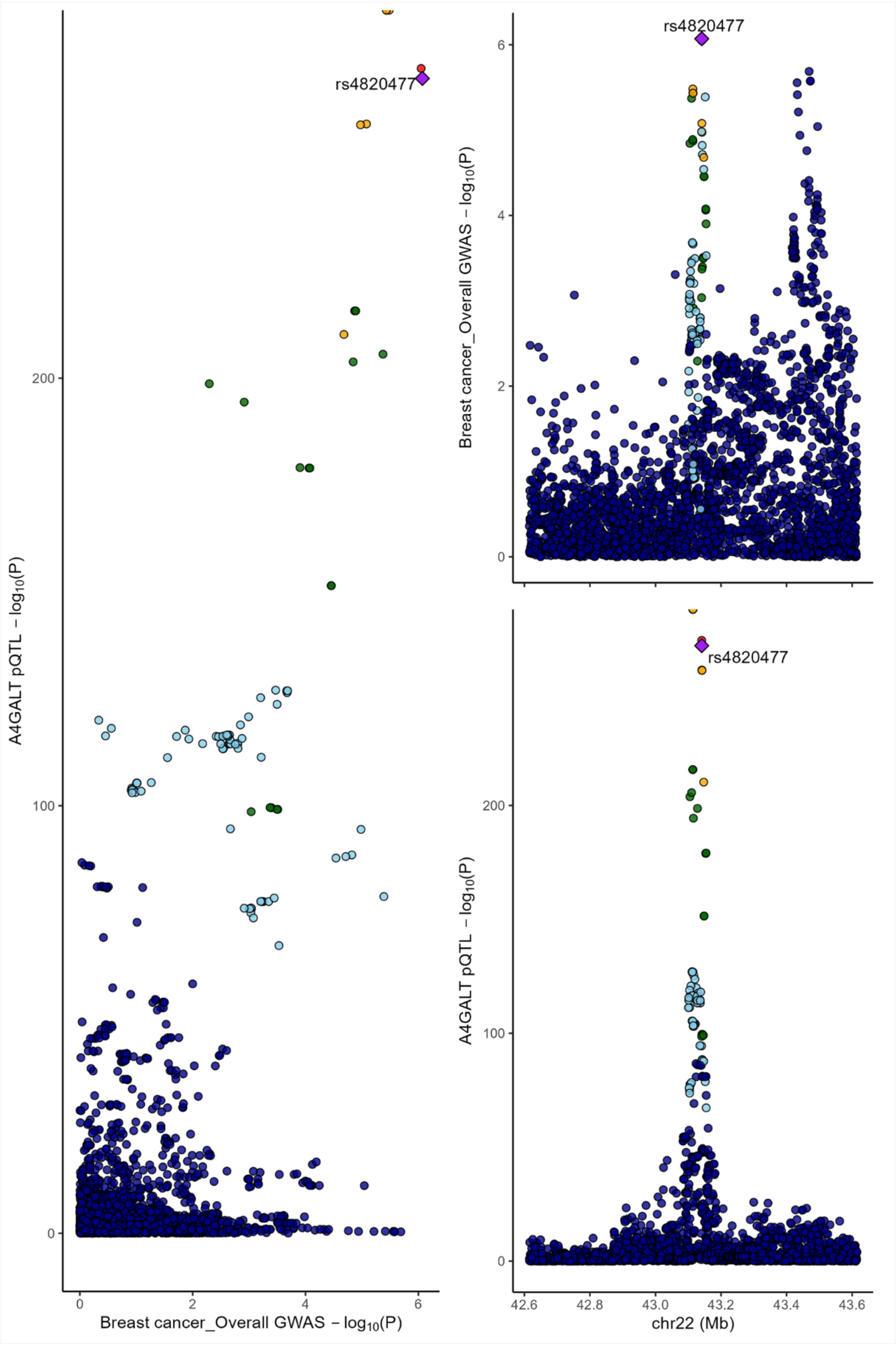

Figure S5 Bayesian colocalization analysis for A4GALT and BC_overall

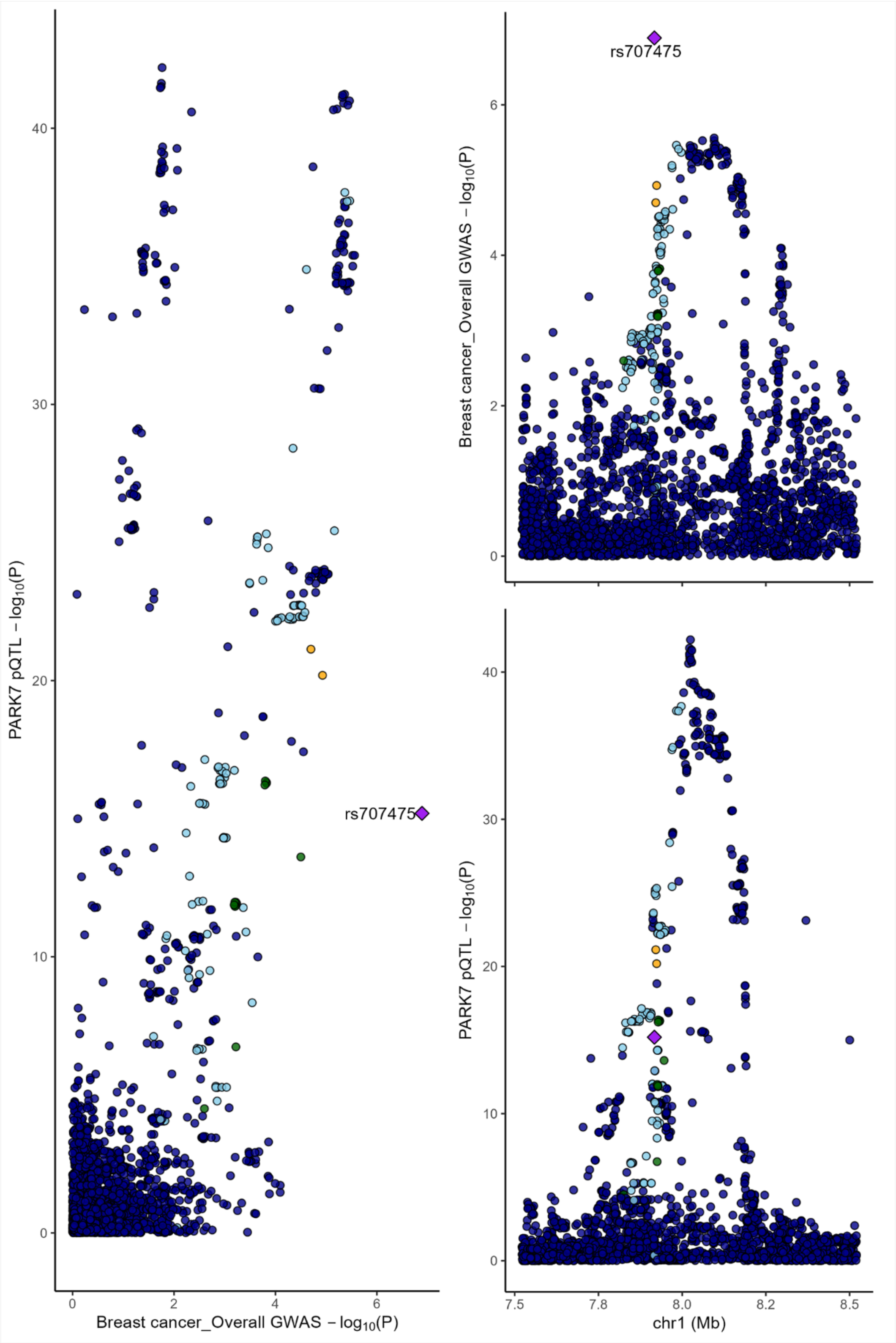

Figure S6 Bayesian colocalization analysis for PARK7 and BC_overall

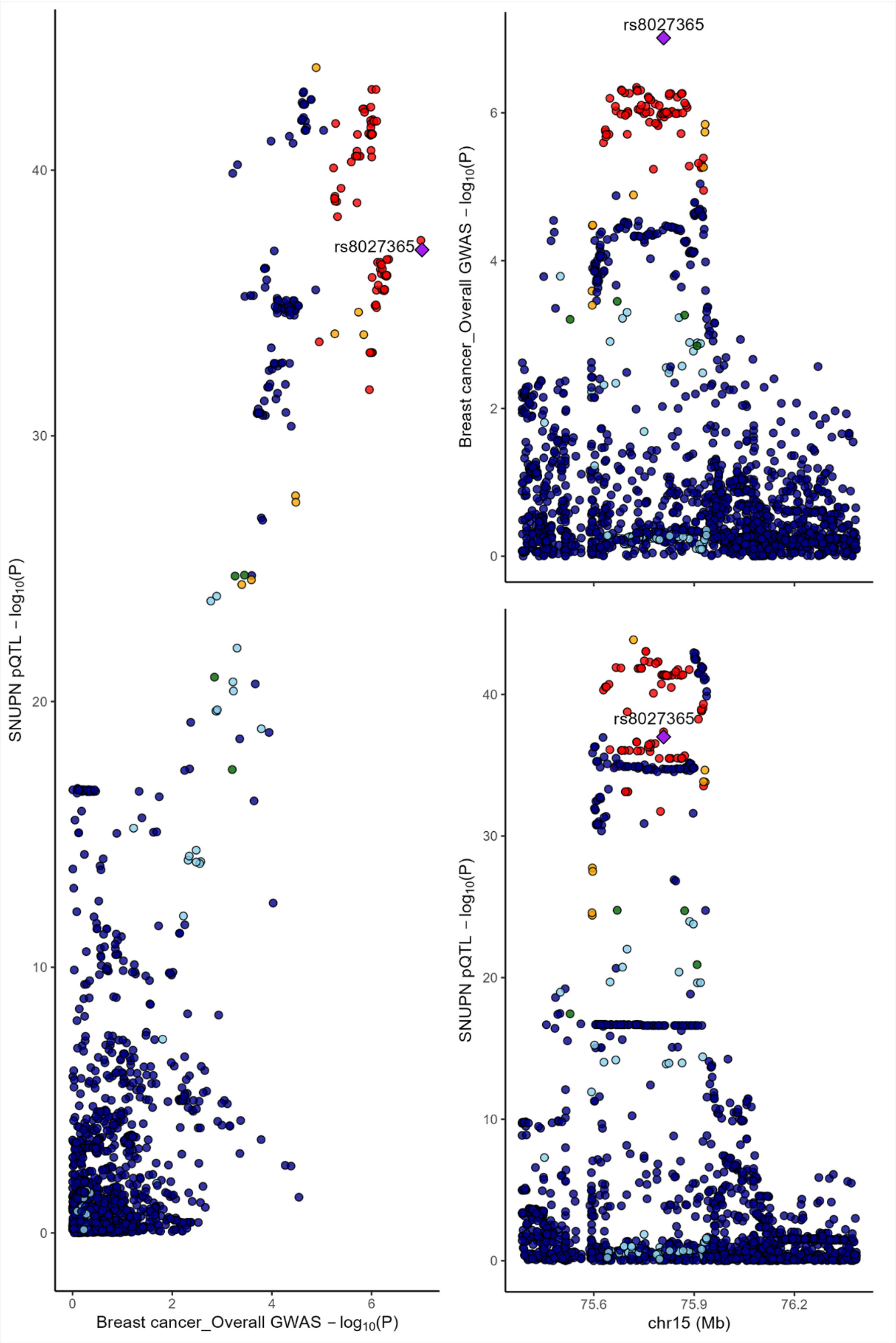

Figure S7 Bayesian colocalization analysis for SNUPN and BC_overall

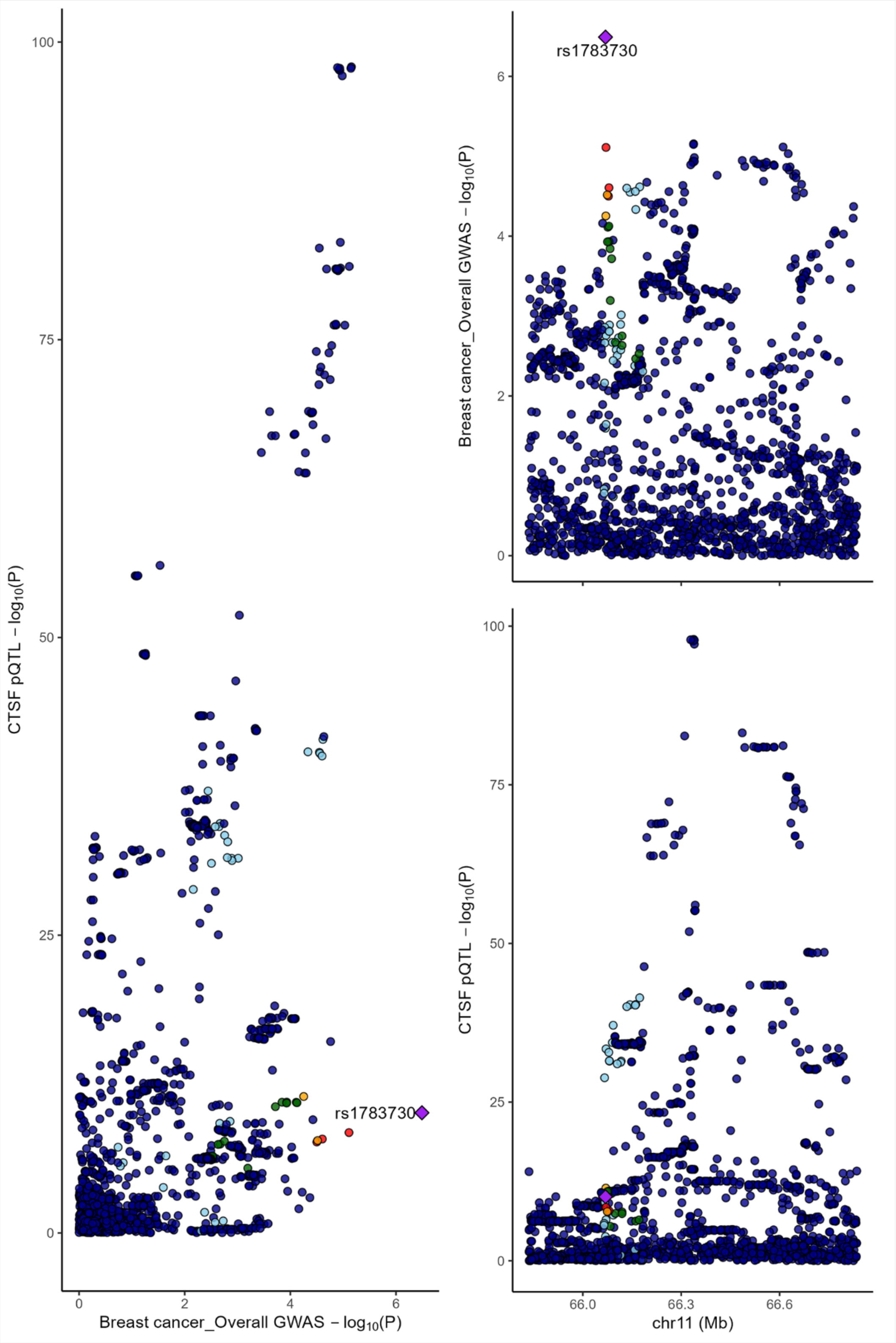

Figure S8 Bayesian colocalization analysis for CTSF and BC_overall

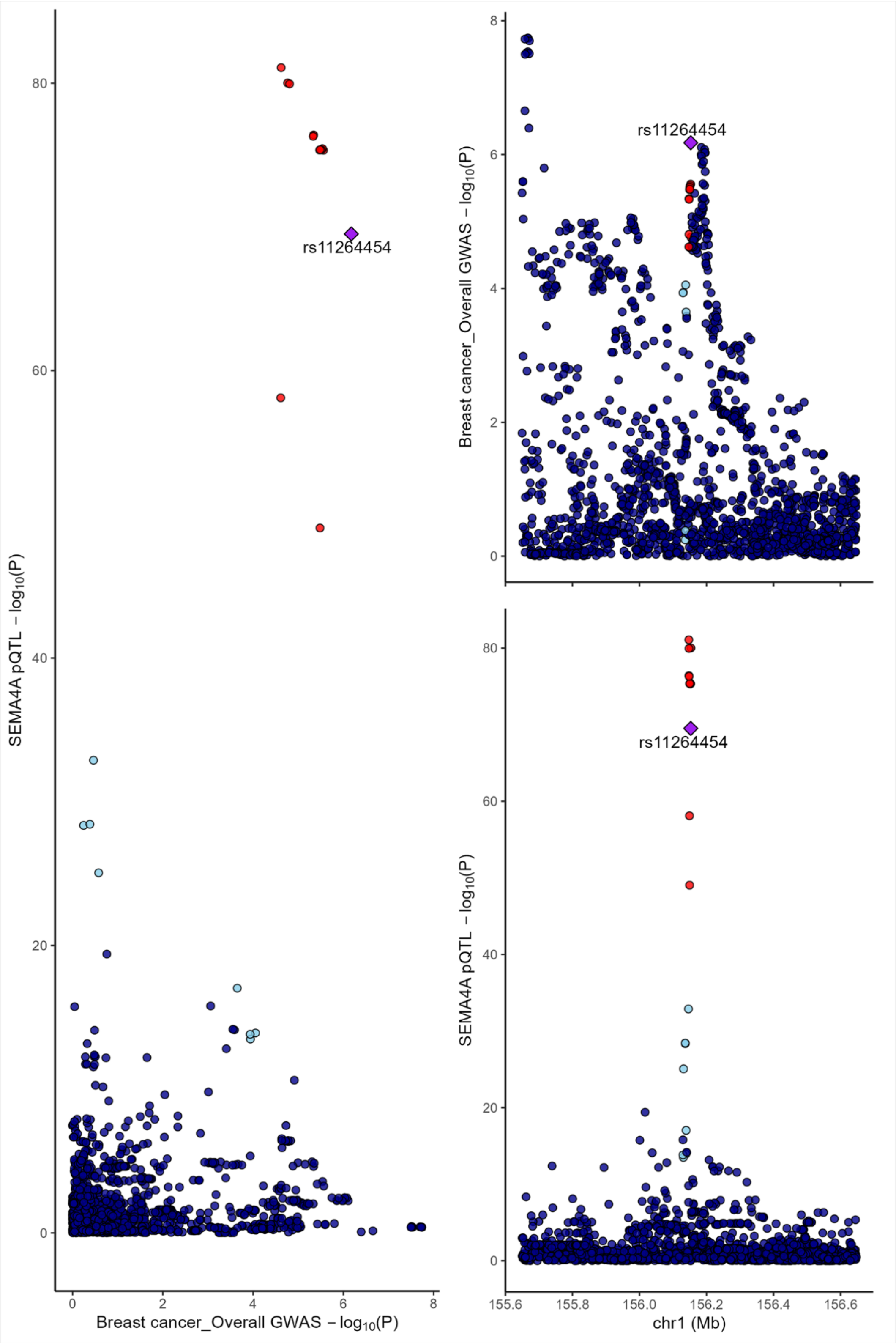

Figure S9 Bayesian colocalization analysis for SEMA4A and BC_overall

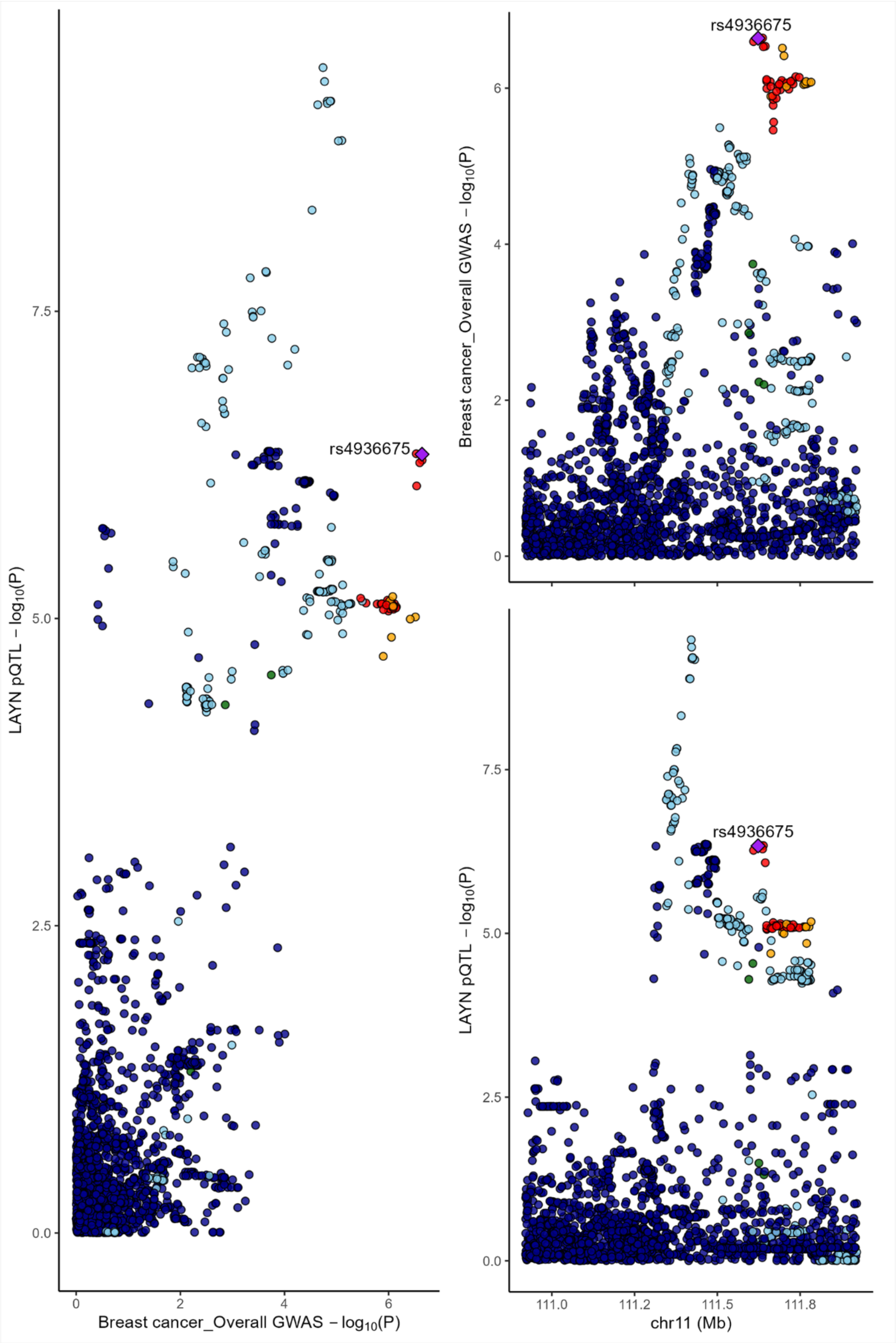

Figure S10 Bayesian colocalization analysis for LAYN and BC_overall

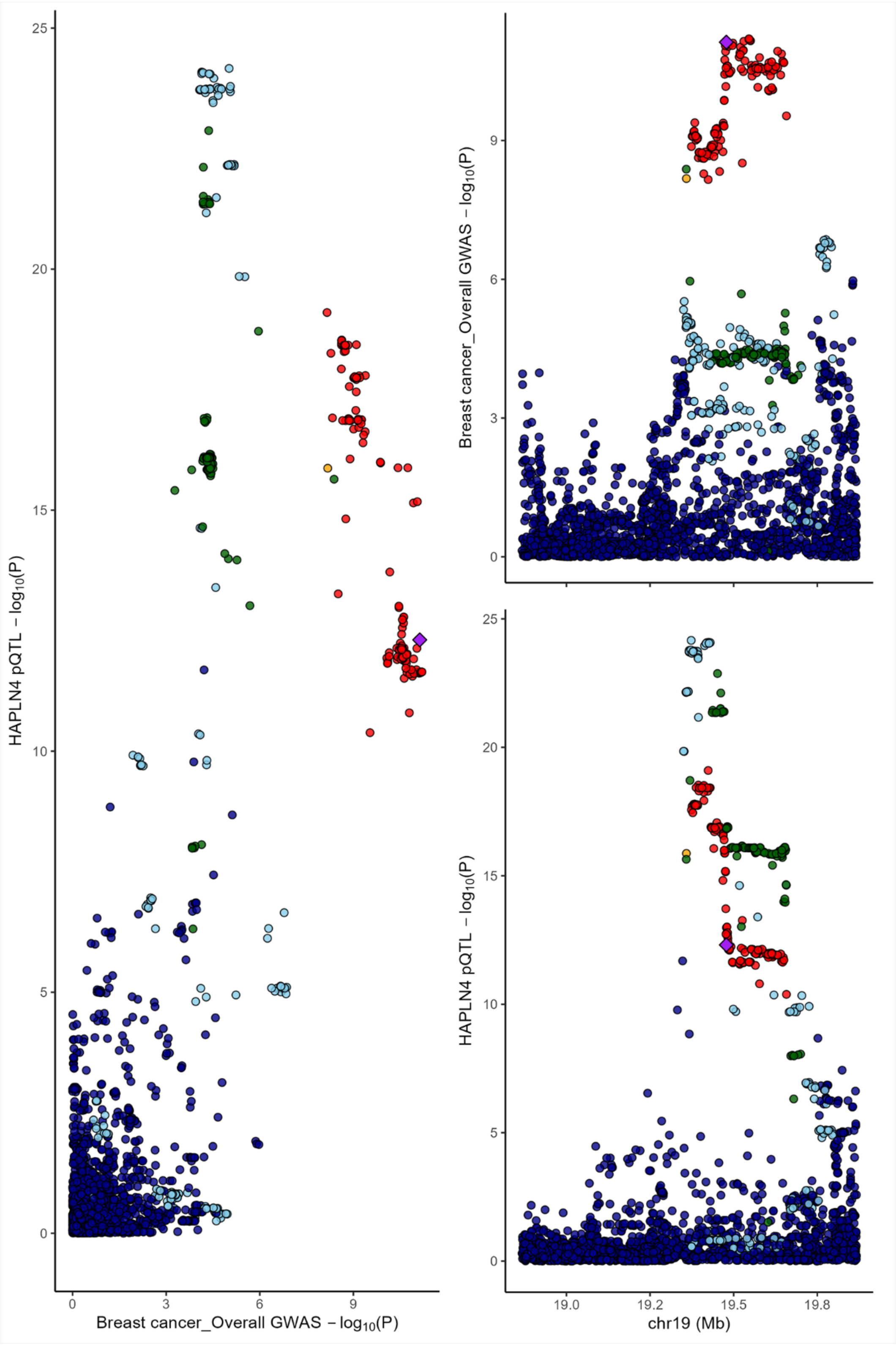

Figure S11 Bayesian colocalization analysis for HAPLN4 and BC_overall

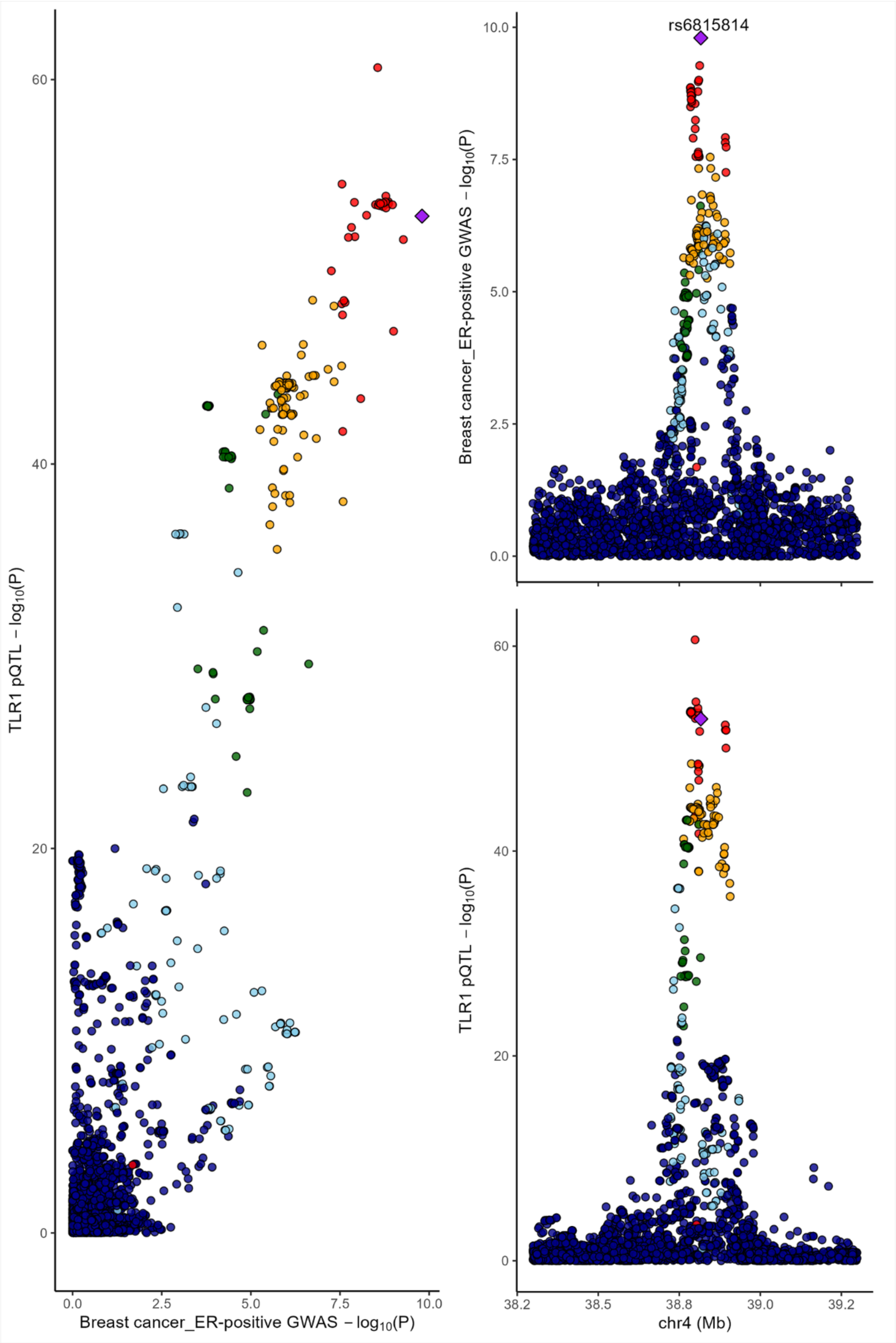

Figure S12 Bayesian colocalization analysis for TLR1 and BC_ER(+)

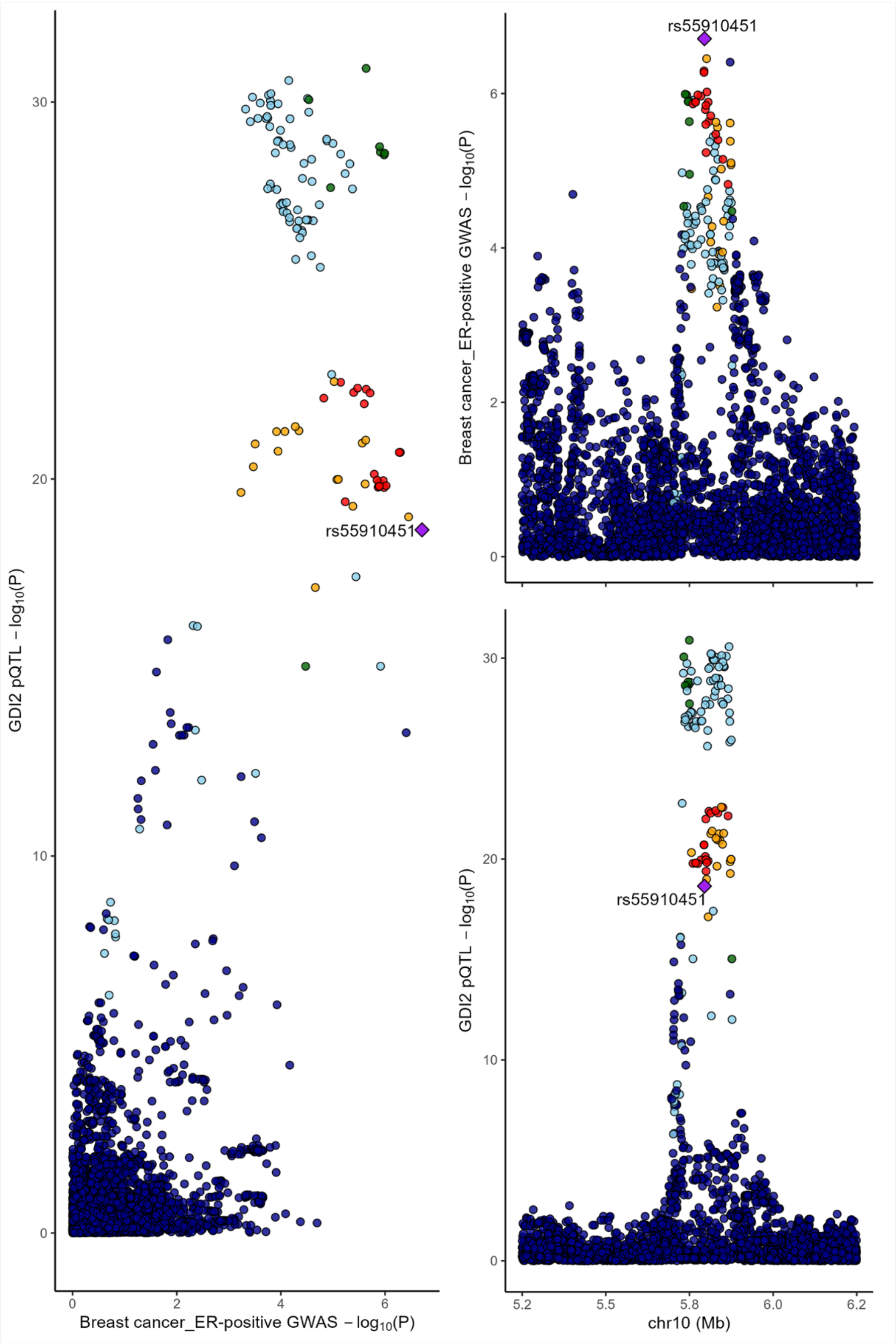

Figure S13 Bayesian colocalization analysis for GDI2 and BC_ ER(+)

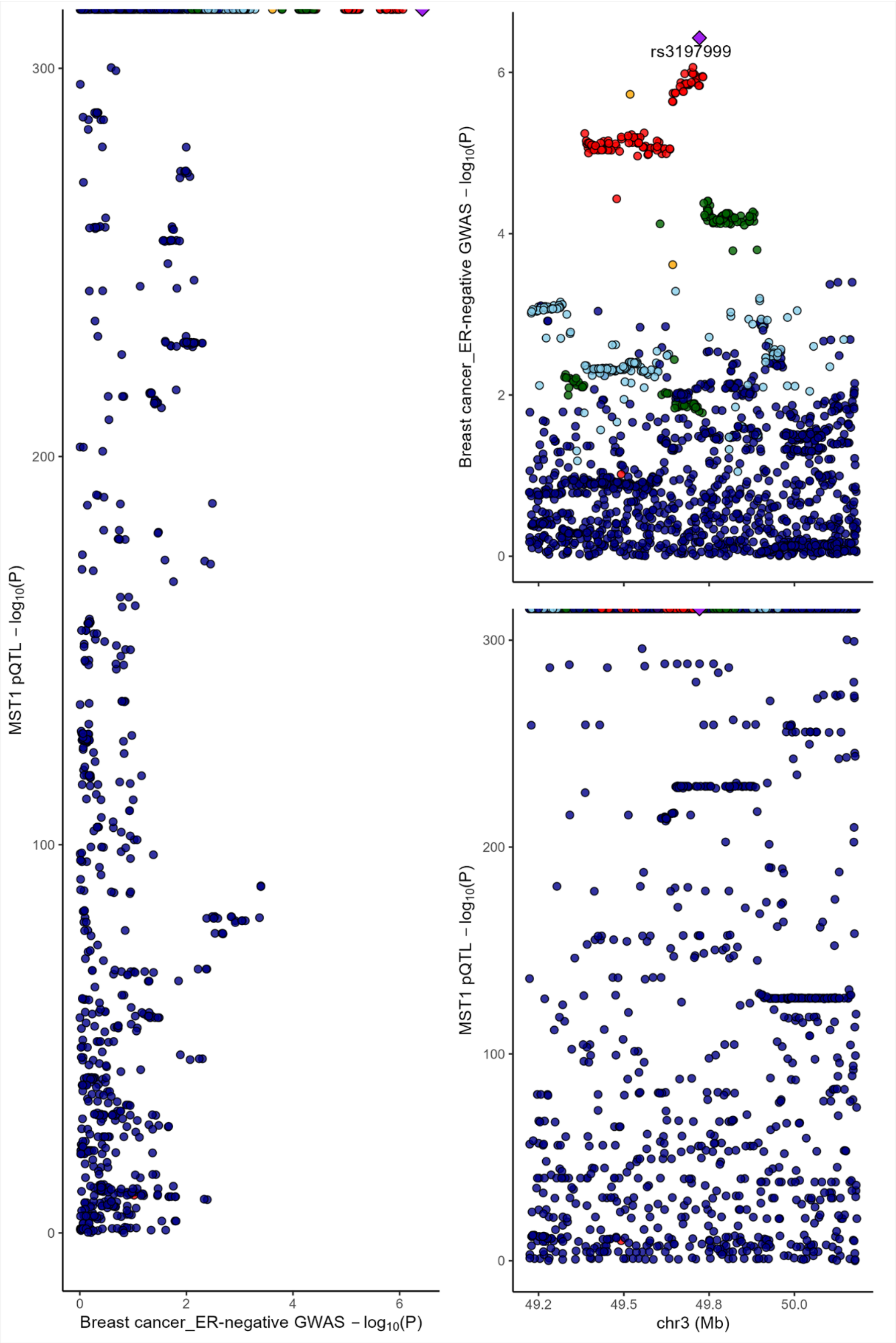

Figure S14 Bayesian colocalization analysis for MST1 and BC_ ER(-)

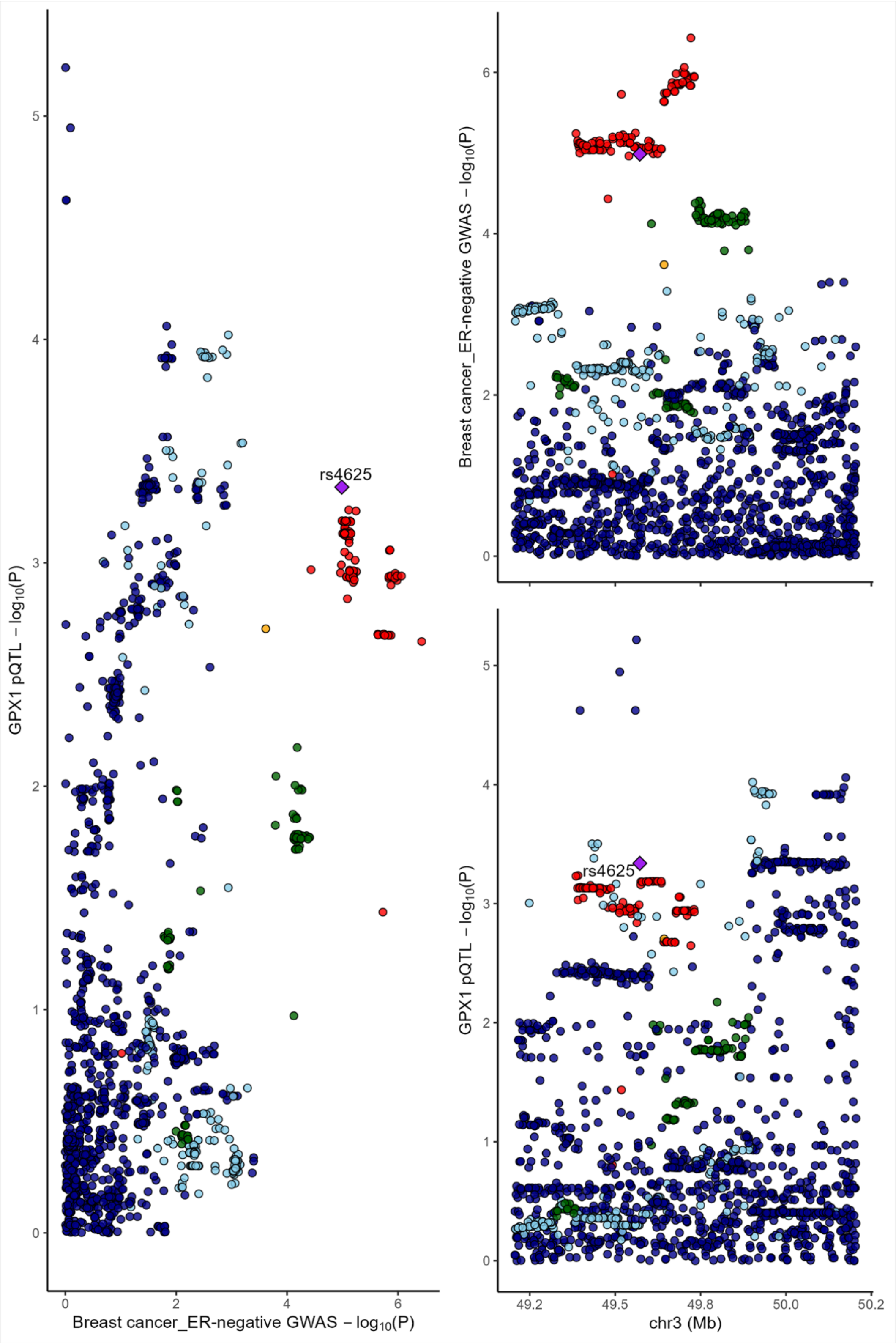

Figure S15 Bayesian colocalization analysis for GPX1 and BC_ ER(-)

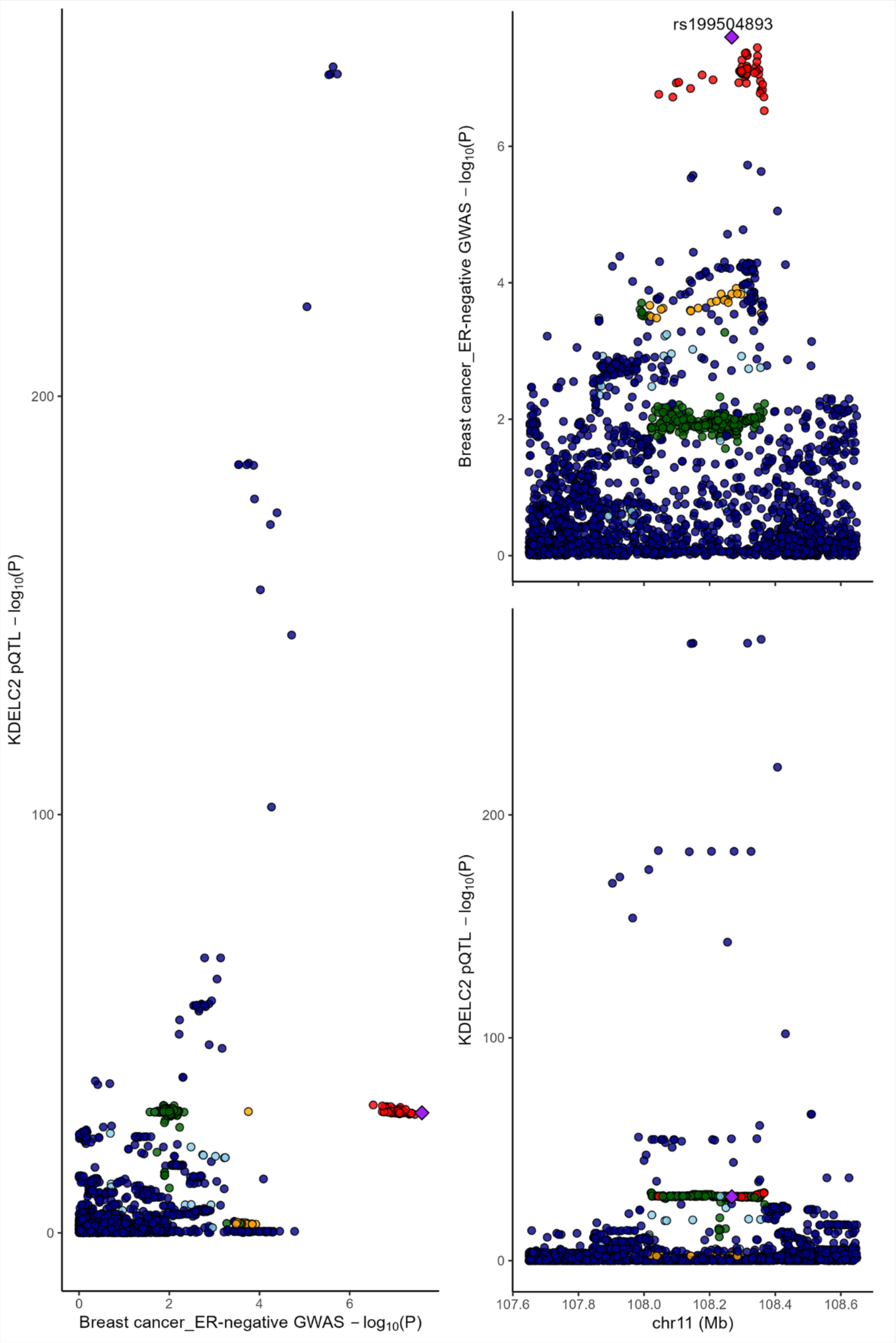

Figure S16 Bayesian colocalization analysis for KDELC2 and BC_ ER(-)

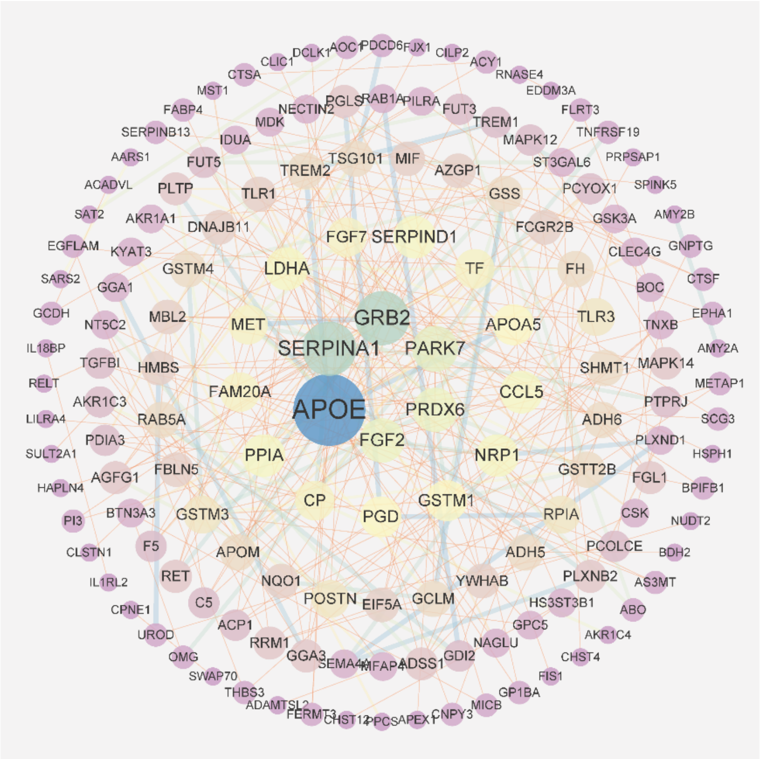

Figure S17 Protein-protein interaction network between suggestive BC_overall-related proteins (P < 0.05) from plasma

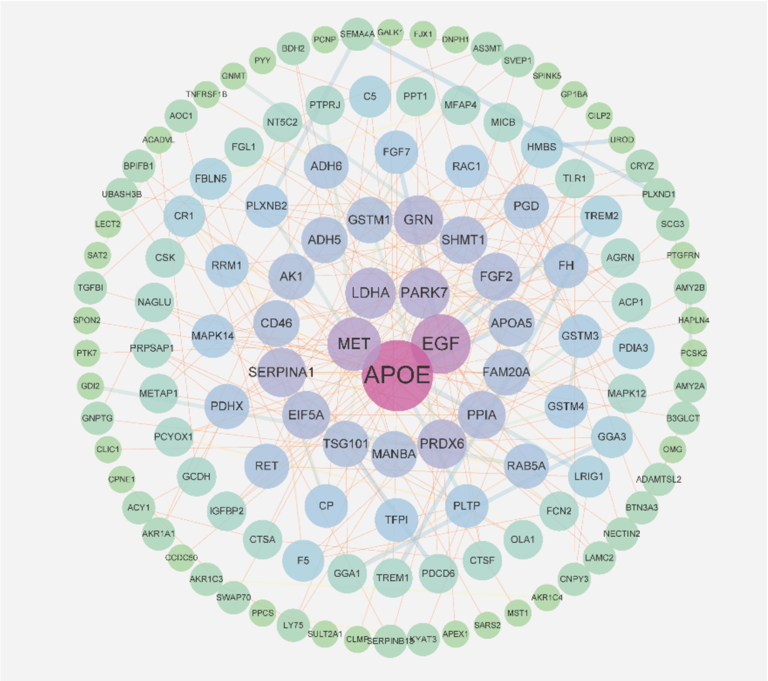

Figure S18 Protein-protein interaction network between suggestive BC_ER(+)-related proteins (P < 0.05) from plasma

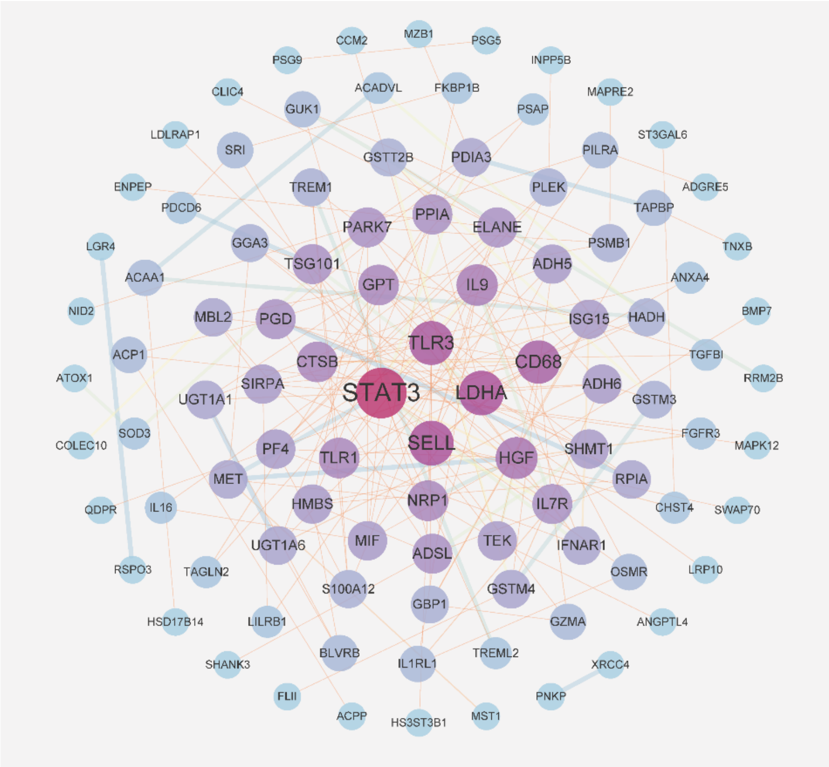

Figure S19 Protein-protein interaction network between suggestive BC_ER(-)-related proteins (P < 0.05) from plasma

Outcome	Protein	Replication analysis(significant) OR(95% CI)	Replication analysis(significant) pval	Replication analysis(same) OR(95% CI)	Replication analysis(same) pval
BC_overall	TLR1	1.457(1.311,1.619)	2.83E-12	1.457(1.311,1.619)	2.83E-12
	PDCD6	1.554(1.274,1.896)	1.39E-05	1.895(1.489,2.412)	2.03E-07
	RCL	1.295(1.146,1.464)	3.47E-05	Removing the SNP for incompatible alleles	
	A4GALT	0.911(0.876,0.948)	3.40E-06	0.913(0.878,0.949)	3.97E-06
	PARK7	0.844(0.734,0.970)	1.68E-02	0.765(0.682,0.858)	4.69E-06
	SNUPN	0.797(0.719,0.882)	1.23E-05	0.790(0.713,0.875)	5.78E-06
	CTSF	1.187(1.101,1.280)	7.53E-06	1.175(1.094,1.262)	1.03E-05
	GCDH	Lack of data		Lack of data	
	SEMA4A	1.179(1.093,1.272)	2.21E-05	1.189(1.100,1.286)	1.37E-05
	LAYN	1.719(1.343,2.201)	1.70E-05	1.719(1.343,2.201)	1.70E-05
	HAPLN4	1.401(1.206,1.627)	1.00E-05	1.377(1.188,1.597)	2.14E-05
BC_ER（+）	TLR1	1.462(1.290,1.657)	2.65E-09	1.462(1.290,1.657)	2.65E-09
	GDI2	Lack of data		0.686(0.587,0.802)	2.24E-06
BC_ER（-）	MST1	1.051(1.030,1.073)	1.23E-06	1.055(1.033,1.077)	3.98E-07
	GPX1	Lack of data		8.709(3.647,20.794)	1.09E-06
	KDELC2	1.217(1.122,1.320)	2.29E-06	1.208(1.117,1.308)	2.60E-06

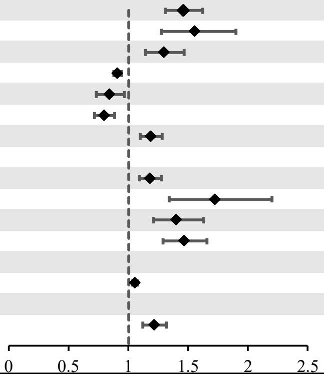

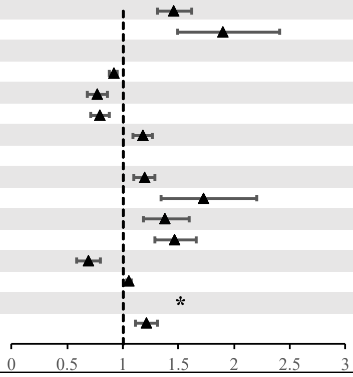

Figure S20 Replication of causal relationship between prioritized proteins and breast cancer

* symbolizes instances where exceptionally large numerical values disrupt the aesthetic appeal of the forest plot, necessitating its use to maintain visual clarity.

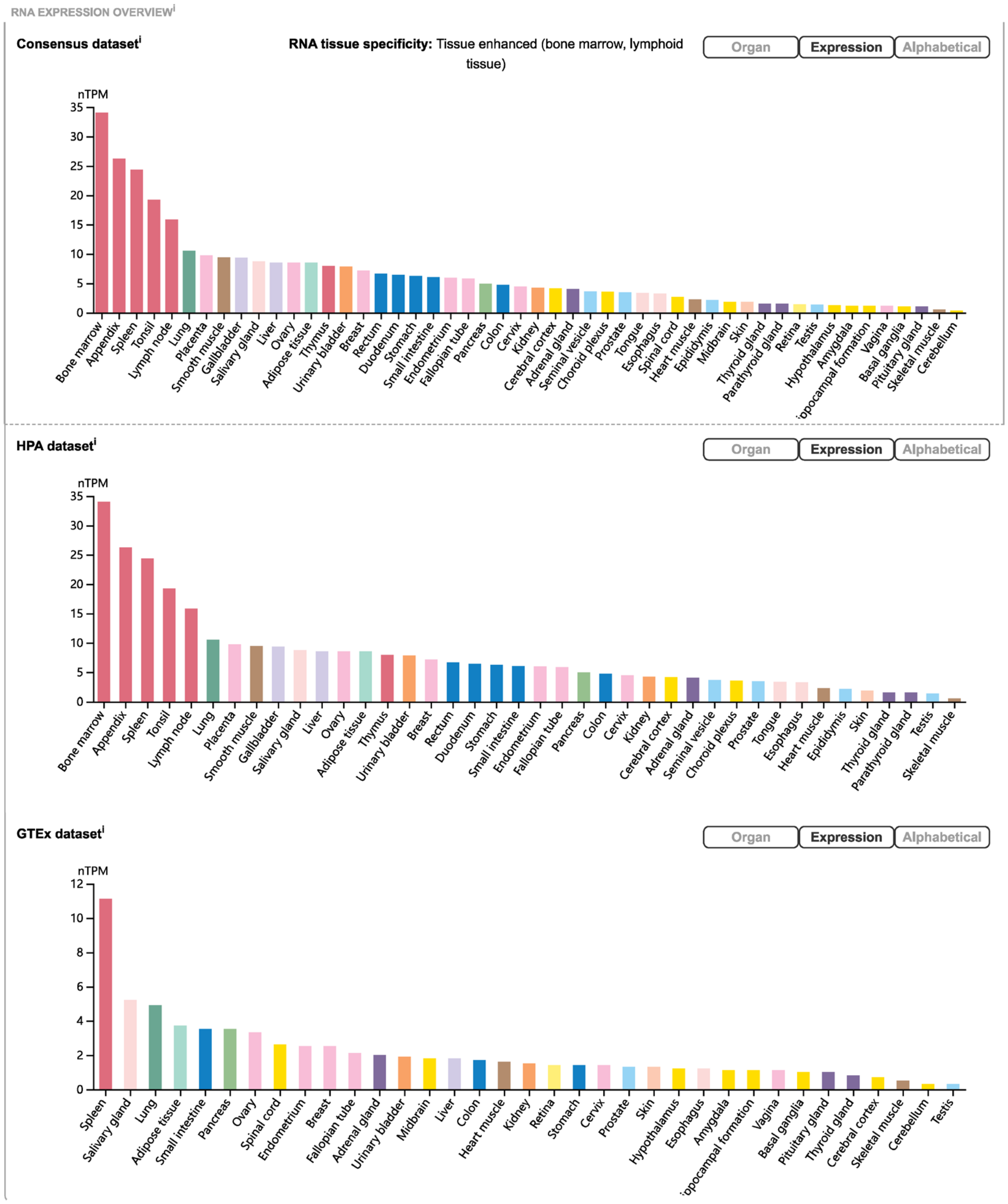

Figure S21 The mRNA expression profile of TLR1 across human tissues (The human protein atlas, https://www.proteinatlas.org/).

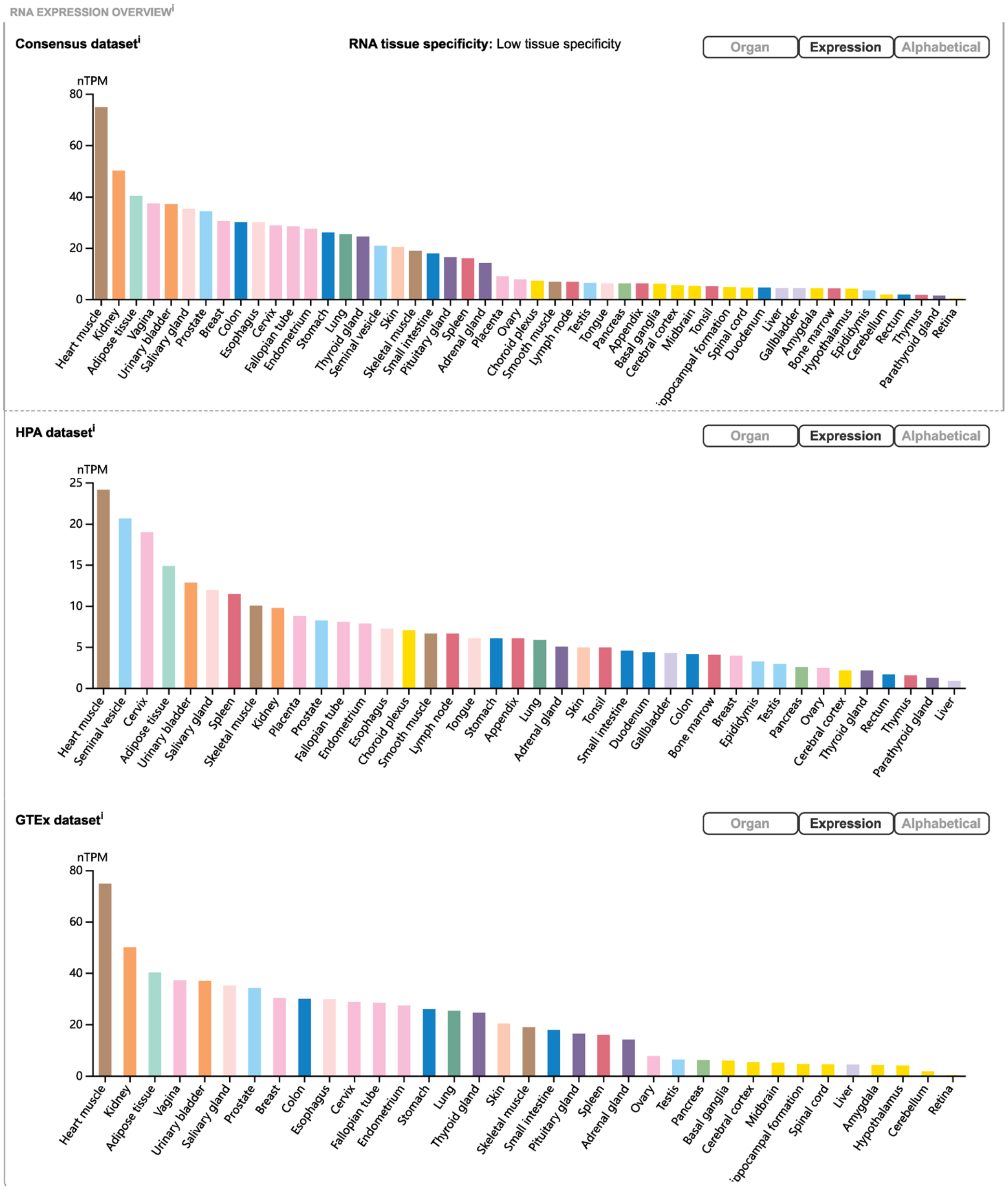

Figure S22 The mRNA expression profile of A4GALT across human tissues (The human protein atlas, https://www.proteinatlas.org/).

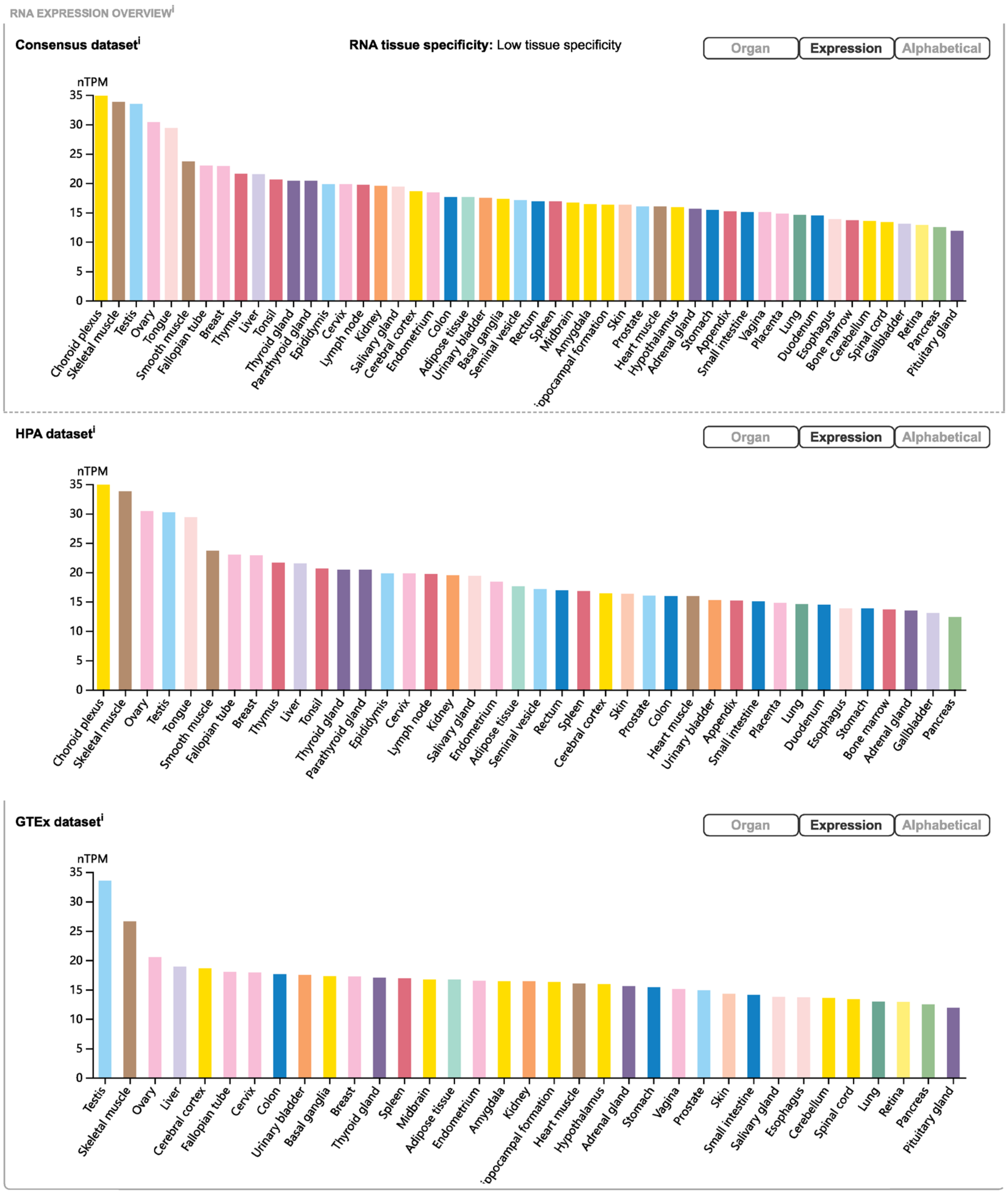

Figure S23 The mRNA expression profile of SNUPN across human tissues (The human protein atlas, https://www.proteinatlas.org/).

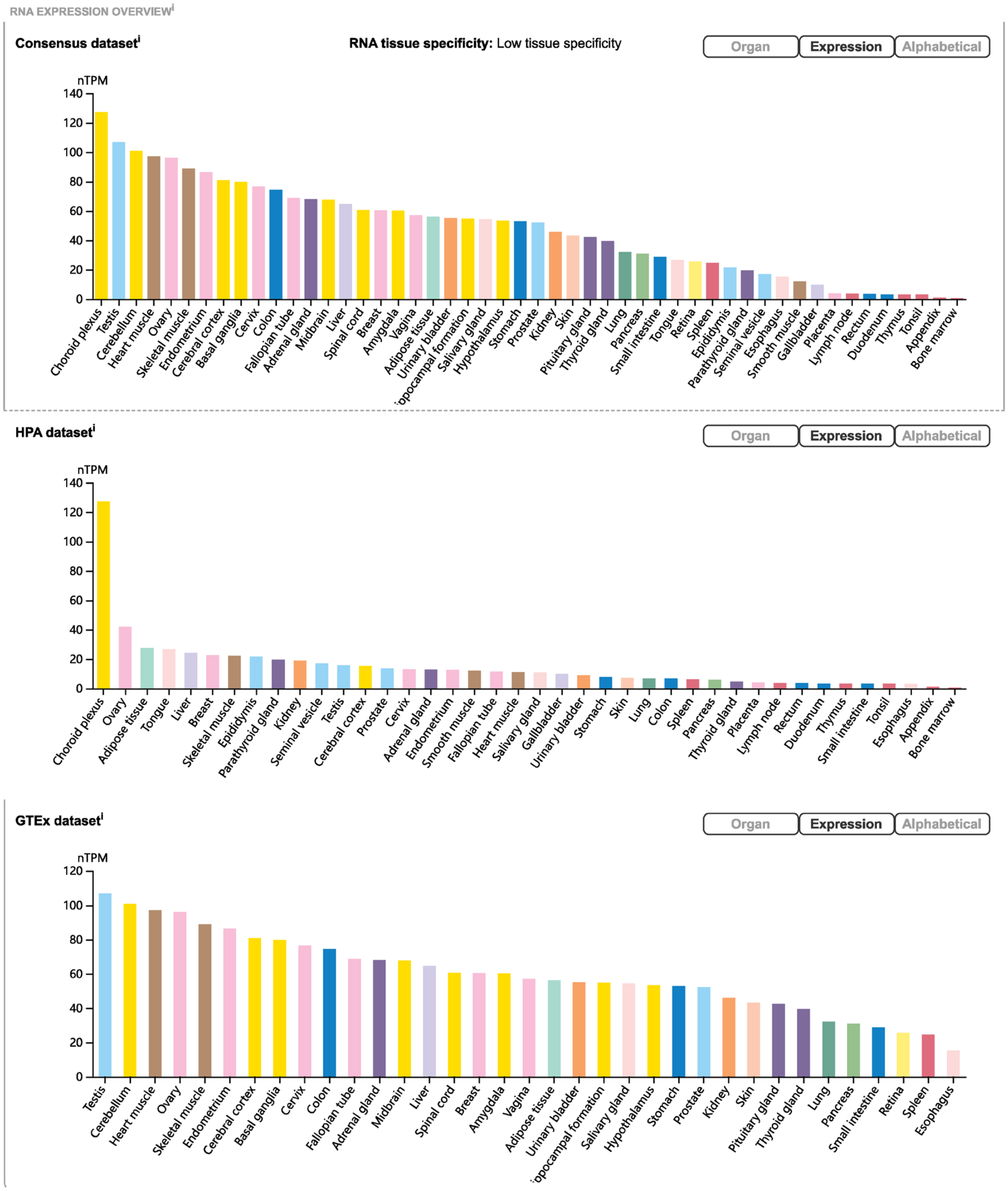

Figure S24 The mRNA expression profile of CTSF across human tissues (The human protein atlas, https://www.proteinatlas.org/).
